# Effects of dialkoxybenzenes against *Varroa destructor* and identification of 1-allyloxy-4-propoxybenzene as a promising acaricide candidate

**DOI:** 10.1038/s41598-023-38187-6

**Published:** 2023-07-11

**Authors:** Soniya Dawdani, Marissa O’Neill, Carlos Castillo, Jorge E. Macias Sámano, Heather Higo, Abdullah Ibrahim, Stephen F. Pernal, Erika Plettner

**Affiliations:** 1 Department of Chemistry, Simon Fraser University, 8888 Univ. Dr., Burnaby, BC V5A 1S6 Canada; 2 Agriculture and Agri-Food Canada, Beaverlodge Research Farm, P.O. Box 29, Beaverlodge, AB T0H 0C0 Canada

**Keywords:** Agroecology, Entomology, Drug discovery and development

## Abstract

The honey bee is responsible for pollination of a large proportion of crop plants, but the health of honey bee populations has been challenged by the parasitic mite *Varroa destructor*. Mite infestation is the main cause of colony losses during the winter months, which causes significant economic challenges in apiculture. Treatments have been developed to control the spread of varroa. However, many of these treatments are no longer effective due to acaricide resistance. In a search of varroa-active compounds, we tested the effect of dialkoxybenzenes on the mite. A structure–activity relationship revealed that 1-allyloxy-4-propoxybenzene is most active of a series of dialkoxybenzenes tested. We found that three compounds (1-allyloxy-4-propoxybenzene, 1,4-diallyloxybenzene and 1,4-dipropoxybenzene) cause paralysis and death of adult varroa mites, whereas the previously discovered compound, 1,3-diethoxybenzene, which alters host choice of adult mites in certain conditions, did not cause paralysis. Since paralysis can be caused by inhibition of acetylcholinesterase (AChE), a ubiquitous enzyme in the nervous system of animals, we tested dialkoxybenzenes on human, honey bee and varroa AChE. These tests revealed that 1-allyloxy-4-propoxybenzene had no effects on AChE, which leads us to conclude that 1-allyloxy-4-propoxybenzene does not exert its paralytic effect on mites through AChE. In addition to paralysis, the most active compounds affected the ability of the mites to find and remain at the abdomen of host bees provided during assays. A test of 1-allyloxy-4-propoxybenzene in the field, during the autumn of 2019 in two locations, showed that this compound has promise in the treatment of varroa infestations.

## Introduction

Many flowering plants rely on insect pollinators to produce fruits, nuts, and seeds^1^. Of these pollinators, the honey bee *Apis mellifera* L. remains the most ubiquitous pollinator of plants which produce food for human consumption^2^. It is estimated that the production of one third of the food supply, as well as fibers and oils, is influenced by the pollination of honey bees and other pollinators^1^. Declines in honey bee populations have been observed in many places worldwide, and in some cases these have been attributed to the collapse of colonies^3^. There are many causes for mortality or morbidity of bee colonies, including bee exposure to pesticides, viruses, bacterial and fungal infections, and parasites^4^.

Of all the factors responsible for bee colony mortality or poor performance, the parasitic mite *Varroa destructor* Anderson and Trueman, commonly referred to as “varroa”, causes the greatest amount of damage and economic cost^5^. Varroa not only feed on the fat body tissues of bees^6,7^, but also transmit viral infections^8,9^. Varroa females time their reproduction with the development of new bees in a hive. They feed on adult nurse bees (which take care of developing brood) and move into brood cells, 15–20 h prior to cell capping for worker brood and 40–50 h for drone brood^9,10^. Varroa then produce one male and several female offspring which mate in the capped cell. Since the mites impact bees at different times during their life cycle, they produce various negative effects which are together referred to as varroosis^5^. Their rapid life cycle can quickly lead to a strong infestation which is capable of crippling a wintering colony within several weeks^11^. In Canada and the US, varroa infestation has been observed to be a major cause of losses of colonies during winter^12,13^.

Many compounds have been evaluated for their efficacy in treating honey bee colonies against varroa, including essential oils and organic acids (oxalic and formic), as well as numerous synthetic acaricides, including pyrethroids, organophosphates and formamidines^9,14–16^. Thymol is a monoterpene that is widely used to treat hives against varroosis^15,17^. These treatments vary in effectiveness, as well as cost and the labour associated with their administration^17,18^. Some treatments (such as essential oils) work most effectively in conjunction with other compounds, and their efficacy depends on temperature, humidity, and the population of bees in the hive^14^. Furthermore, some treatments for varroa can negatively affect bees, and they or their degradation products can leave residues in wax or honey^19–22^.

**Figure 1:**
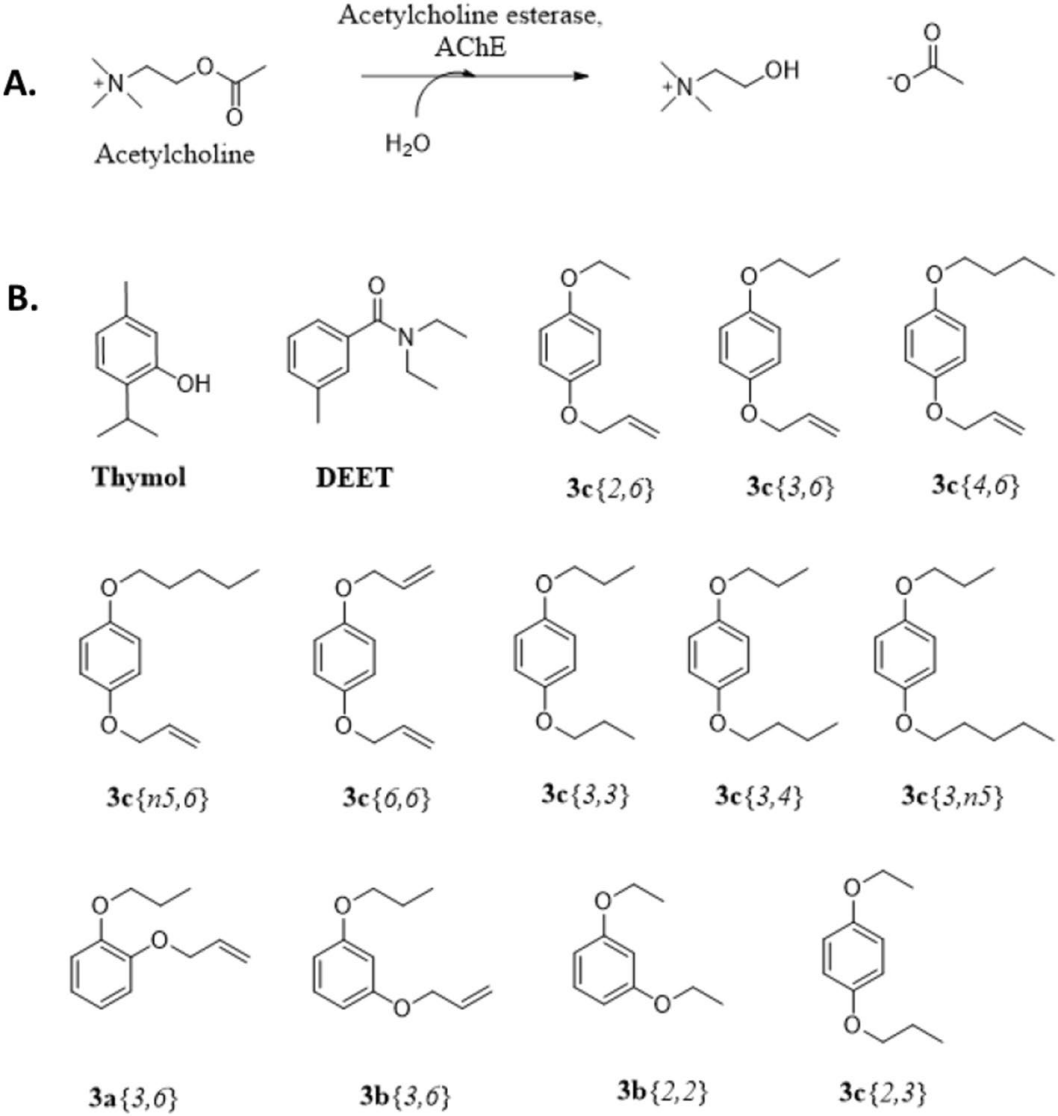
**(A)** Acetylcholinesterase-catalyzed reaction. (**B)** Compounds tested in the structure–activity survey.

The palette of substances currently approved for treatment of varroosis is limited and shrinking due to the development of resistance against the most widely used acaricides. Thus, the acaricide fluvalinate (sold under the name of Apistan^®^), which had low toxicity to bees but was effective against the mites^17^, is no longer useful, due to the mites developing resistance against this compound through mutations in the target site, a voltage-gated sodium channel^23–27^. Another acaricide against which varroa have developed resistance is coumaphos^25,28,29^, an organophosphate pro-insecticide that is converted to a metabolite (coroxon) that targets the synaptic enzyme acetylcholinesterase (AChE), which catalyzes the breakdown of acetylcholine to choline and acetate (Fig. 1A). This process is essential in both the peripheral and central nervous system of animals; therefore, disrupting it can lead to problems with neurotransmission, behaviour, and locomotion^30,31^. Interestingly, resistance of varroa to coumaphos has different mechanisms in various places. In populations of resistant varroa mites in Greece, it was shown to be caused by a downregulation of the activating cytochrome P450 in the mites, CYP4EP4. Thus, resistant mites produce less coroxon than susceptible ones, upon exposure to coumaphos^29^. In the US, coumaphos-resistant mites were found to have AChE that was 50× less sensitive to coroxon inhibition than susceptible mites^32^. Resistance to the widely used acaricide amitraz is variable, with mites from some places showing no resistance^33^ and mites from other locations showing some resistance^24,34,35^, though the underlying mechanism for that resistance is not known.

Alterations in the mites that lead to resistance carry a cost in fitness, thus, non-use of one particular acaricide for even one treatment cycle can lead to a decrease in the proportion of resistant individuals in a colony^36,37^, whereas a single treatment can lead to an increase^36^. It is therefore important to find new compounds which selectively target varroa and which can be alternated with other treatments in integrated pest management (IPM) routines.

New treatments against varroa mites are being developed in various laboratories. Riva et al. (2019) have examined the AChE of varroa and docked a series of thiazolo benzodiazepine thiones into the active site. They found two congeners that inhibited the varroa enzyme ~ 60% but the bee AChE only 15–20%. In cages, these compounds caused varroa mortality, though at approximately 10× lower intensity than amitraz^38^. In another effort, a voltage-gated chloride channel blocker was studied in the lab and in the field. The most active chloride channel blocker showed ~ 70% efficacy against *tau*-fluvalinate- or coumaphos-resistant mites^32^. Bahreini et al*.* (2020) have screened a panel of known miticides registered for use on crops in a laboratory assay and found fenpyroximate, fenazaquin and etoxazole as leading candidates^39^. They tested fenazaquin and fenpyroximate in a field assay, in which 3-frame replicated colonies were treated with variable doses of these compounds for 42 days, followed by a final treatment with oxalic acid. Fenazaquin had 80% efficacy whereas fenpyroximate had 68%^40^.

Our group previously synthesized a set of compounds which showed promise in their ability to change the host choice preference of varroa from nurse bees to forager bees^41^. One of the most active compounds in that series was 1,3-diethoxybenzene, **3b**{*2,2*}^30^ (Fig. 1B). We have previously found that the related compound, 1-allyloxy-4-propoxybenzene, **3c**{*3,6*} (Fig. 1B), is a feeding deterrent against Lepidoptera^42^ and a mosquito repellent as strong as DEET, *N,N*-diethyl-*m*-toluamide^43^. Therefore, we tested this compound against varroa and found that compound **3c**{*3,6*} prevents mites from choosing a host bee by slowing the mite’s movements. This paralysis was observed to lead to higher-than-normal mite mortality in those assays, whereas bees exposed orally to high doses of **3c**{*3,6*} did not show acute symptoms^44^. A similar delay in host choice of varroa was observed previously with DEET^45^, which has been shown to inhibit AChE in insects and mammals^46,47^.

In this study we investigated the structure–activity relationship of compound **3c**{*3,6*} and closely related compounds, such as 1,4-diallyloxybenzene **3c**{*6,6*}, 1,4-dipropoxybenzene **3c**{*3,3*} or the positional isomers of **3c**{*3,6*}, 1-allyloxy-2-propoxybenzene **3a**{*3,6*} and 1-allyloxy-3-propoxybenzene **3b**{*3,6*} (Fig. 1B), on the movement and survival of mites in laboratory assays. We included thymol as a positive control because of its known acaricidal effects on mites^15,17^ and its structural core (a benzene ring) (Fig. 1B), and we included DEET because of its known inhibition of AChE and its previously detected activity on varroa mites (the delaying of host choice)^45^. We studied dose responses for compounds **3c**{*3,6*} and **3c**{*6,6*}, as well as the time course of the activity of compound **3c**{*3,6*}. We evaluated whether compounds **3c**{*3,6*}, **3c**{*6,6*}, **3b**{*2,2*} and DEET had an inhibitory effect on AChE from varroa *Vd*AChE, humans *h*AChE and bees *Am*AChE, using the Ellman assay (supplementary information, Fig. S1)^48^, which is widely used to monitor the effect of drugs on AChE activity^49^. Having found no major effect of compound **3c**{*3,6*} on AChE, but acaricidal activity against varroa mites, we then tested it in the field in 2019, in two locations: Langley, British Columbia (BC) and Beaverlodge, Alberta (AB).

## Results

### Assays with mites

#### Structure–activity relationship

An initial structure–activity survey was done in 2018 with compounds **3c**{*2,6*}, **3c**{*3,3*}, **3c**{*3,4*}, **3c**{*4,6*}, **3c**{*3,6*} and **3c**{*6,6*}. The compounds were tested at 3 h and 5 h for their ability to affect the host choice made by the mite (Fig. S3A, B) and the paralysis and/or death of the mite (Fig. S3C, D). The compounds did not affect host choice, except **3c**{*2,6*}, which discouraged any choice significantly more (Kruskal–Wallis, *P* < 0.05) than the others, at both 3 h and 5 h. The compounds differed in their ability to paralyze the mites after 3 h of exposure: compounds **3c**{*3,3*} and **3c**{*3,6*} were the most active (Fig. S3C), whereas **3c**{*2,6*} and **3c**{*3,4*} were least active. At 5 h, the numbers of dead mites increased, and all compounds tested showed similar levels of mite paralysis and death (Fig. S3D). Only compound **3c**{*3,6*} differed significantly from the blank at 5 h with regard to paralysis and combined death + paralysis (Kruskal–Wallis, *P* < 0.05).

**Figure 2:**
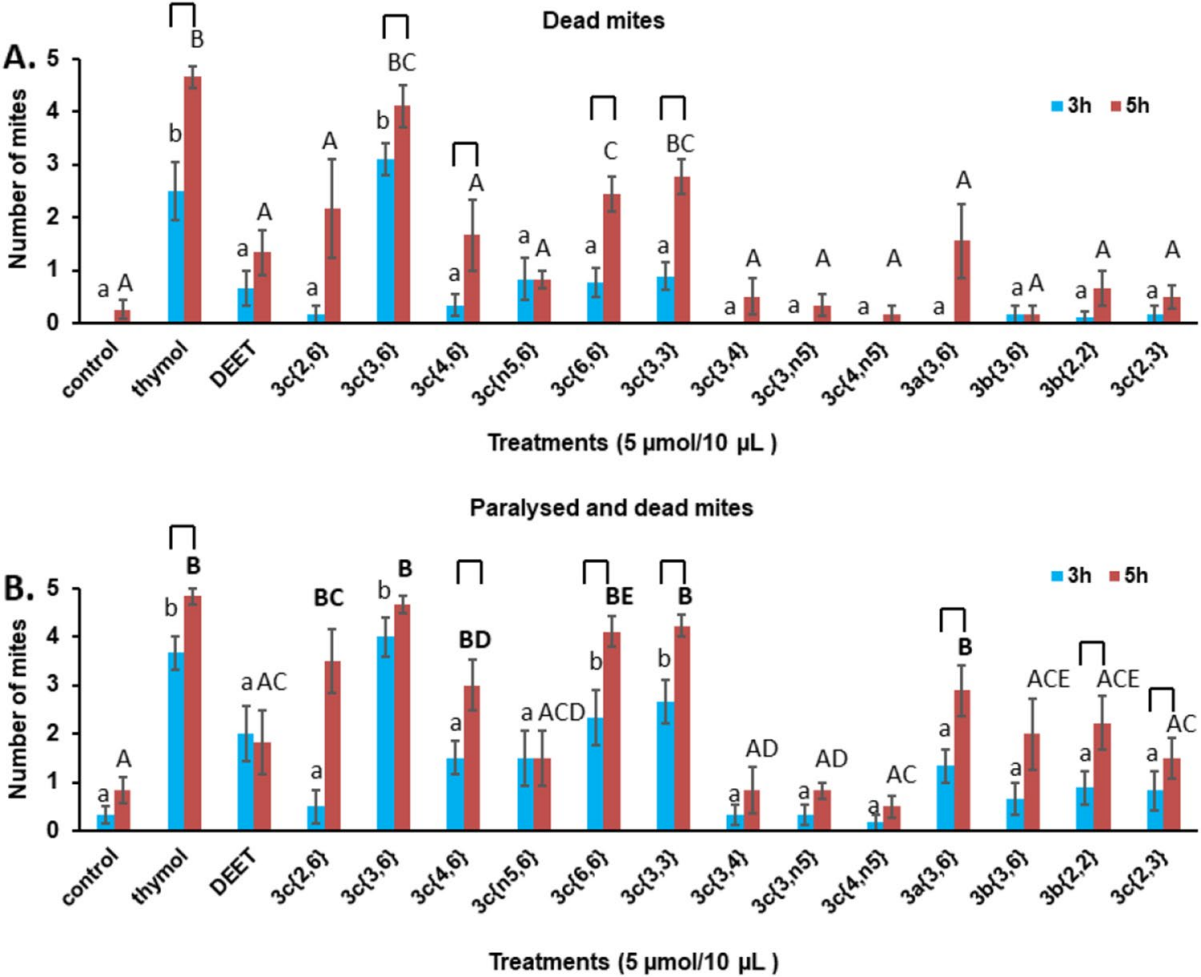
Structure–activity relationship between the 15 compounds tested. The control received only solvent (see text). (**A**) Number of dead mites (out of a total of five mites/replicate), counted 3 h (blue) and 5 h (red) after start of the experiment. (**B**) Total number of paralyzed and dead mites counted 3 h (blue) and 5 h (red) after start of the experiment. Bars represent mean ± S.E. of 6–12 replicates. Letters: comparison between treatments in each time group—ANOVA Tukey, columns marked with different letters differ significantly *P* < 0.05: lower case = 3 h, upper case = 5 h. Brackets above the letters indicate a significant difference between the 3 h and 5 h measurements for that compound (*P* < 0.05).

**Figure 3:**
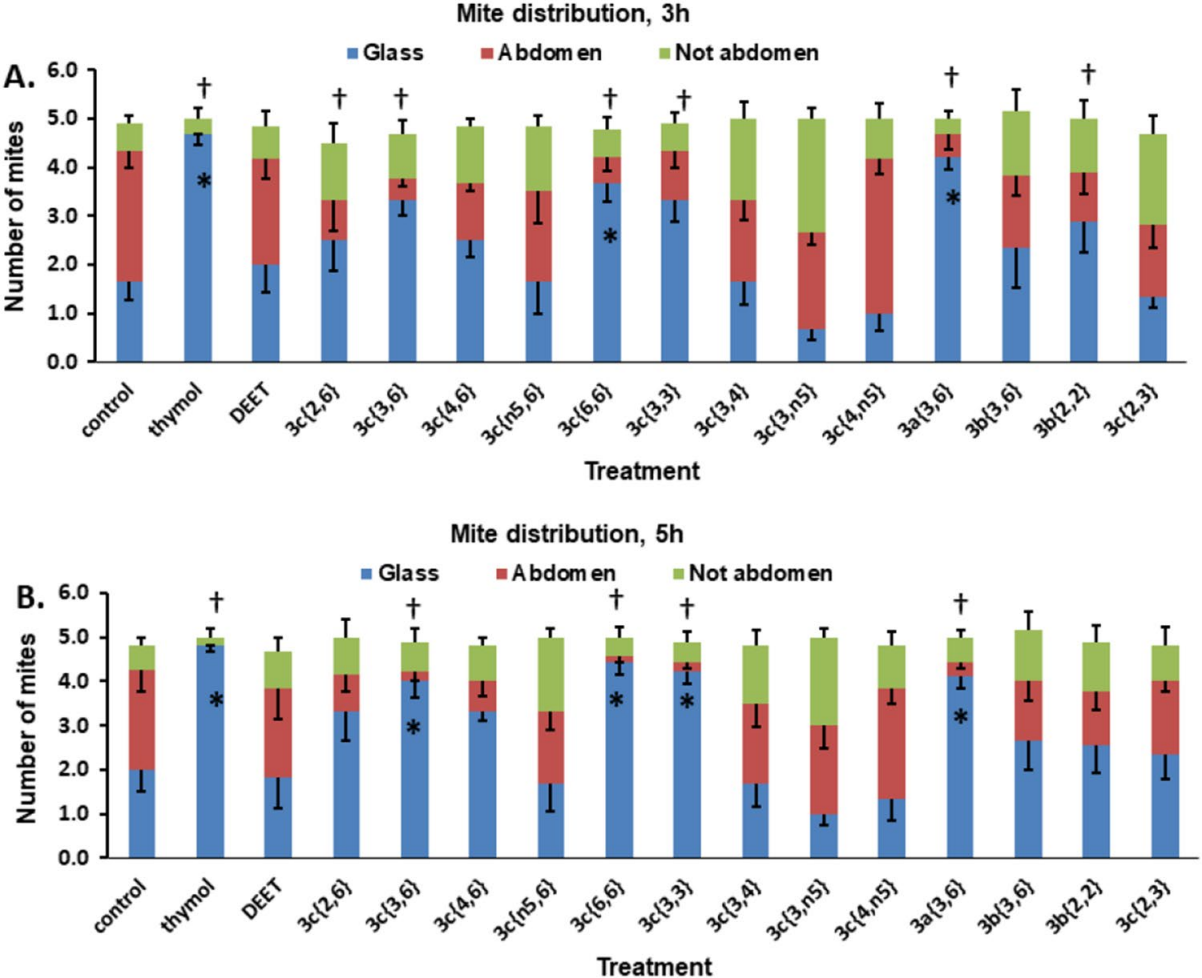
Distribution of mites in the structure–activity experiment. Mites found on the glass are shown in blue, mites found on the abdomen of either bee in the dish are shown in red and mites found on either bee but not on the abdomen are shown in green. Bars represent the mean ± S. E. of 6–12 replicates. Significant differences (*P* < 0.05) between the number of mites on the glass for a compound compared to the control are indicated with a star (*). Significant differences (*P* < 0.05) between the numbers of mites on the abdomen for a compound compared to the control are indicated with a dagger (†).

Based on the initial survey, we expanded the set of compounds in 2020. The structure–activity survey (Fig. 2) was analyzed for the following activities: number of dead mites, the total number of dead and paralyzed mites, the number of mites found on the glass and the number of mites found on the abdomen of either of the bees provided as food. Data are graphed in Figs. 2 and 3, and they are listed in Table S1.

#### Mite death

At 3 h, thymol and **3c**{*3,6*} showed significantly more dead mites than the control or any other compound (Fig. 2A, blue bars). At 5 h, thymol, **3c**{*3,6*}, **3c**{*6,6*} and **3c**{*3,3*} had significantly more dead mites than the control or the remaining compounds (Fig. 2A, red bars). These four active compounds had significantly more dead mites at 5 h than at 3 h.

#### Mite death + paralysis

At 3 h, thymol, **3c**{*3,6*}, **3c**{*6,6*} and **3c**{*3,3*} had significantly more paralyzed + dead mites than the control or the other compounds (Fig. 2B, blue bars). At 5 h, thymol, **3c**{*2,6*}, **3c**{*3,6*}, **3c**{*4,6*}, **3c**{*6,6*}, **3c**{*3,3*} and **3a**{*3,6*} had significantly higher numbers of paralyzed + dead mites than the control (Fig. 2 B, red bars). Among the compounds active at 5 h, thymol, **3c**{*4,6*}, **3c**{*6,6*} **3c**{*3,3*} and **3a**{*3,6*} had more dead + paralyzed mites at 5 h than at 3 h.

Taken together, these results show that thymol and **3c**{*3,6*} were most acaricidal and acted most quickly. Next in terms of acaricidal activity were **3c**{*6,6*} and **3c**{*3,3*}. The least active compounds were **3c**{*2,6*}, **3c**{*4,6*} and **3a**{*3,6*}, their activity being detectable only after 5 h and by taking the total of dead and paralyzed mites. It is of note that DEET and **3b**{*2,2*}, which had been assayed previously against mites (see “Introduction”), were not active in this assay.

#### Mite location on the glass

At 3 h, thymol, **3c**{*6,6*} and **3a**{*3,6*} had significantly higher numbers of mites on the glass than the control or the other compounds (Fig. 3A, blue sections). At 5 h, thymol, **3c**{*3,6*}, **3c**{*6,6*}, **3c**{*3,3*} and **3a**{*3,6*} had significantly higher numbers of mites on the glass than the control or the other compounds (Fig. 3B, blue sections).

#### Mite location on the bee abdomen

At 3 h, thymol, **3c**{*2,6*}, **3c**{*3,6*}, **3c**{*6,6*}, **3c**{*3,3*}, **3a**{*3,6*} and **3b**{*2,2*} had significantly fewer mites on the abdomen of the bees than the control (Fig. 3A, red sections). At 5 h, thymol, **3c**{*3,6*}, **3c**{*6,6*}, **3c**{*3,3*} and **3a**{*3,6*} had significantly fewer mites on the abdomen of the bees (Fig. 3B, red sections).

Taken together, these results suggest that the acaricidal compounds also caused the mites to fail to arrest on the abdomens of the bees provided as food. Compound **3a**{*3,6*} is of note, as it was very active in causing failure to arrest, yet was not very acaricidal. It is also interesting to note that, at 3 h, compound **3b**{*2,2*} caused failure of the mites to arrest on the bee abdomen. Previously, this compound was shown to be among the strongest to alter the host choice of mites between a forager and a nurse^41^. The effect of **3b**{*2,2*} was short-lived, as it was not detected at 5 h. A quantitative structure–activity analysis revealed that compound activity correlated with total accessible surface area (ASA) and with hydrophobic accessible surface area of the compounds (Supplemental Fig. S4). Thus, the smaller the accessible surface area, the more active the compound. There was a modest negative correlation between the log of the octanol water partition coefficient (log P_ow_) and paralysis + death of mites (Fig S4). The congeners with larger alkyl substituents (and larger log P_ow_ values) were less active. There was no correlation with the dipole moment of the compounds. Among the dialkoxybenzenes, the most active compounds were *para* substituted and had propoxy or allyloxy substituents. The activity of the compounds also did not correlate with their volatility, as determined in this study (Supplemental Fig. S5). The most salient comparison is for thymol (most volatile) with **3c**{*3,6*} (20× less volatile than thymol), both of which had equal acaricidal activity.

#### Time course of acaricidal activity and mite distribution

**Figure 4:**
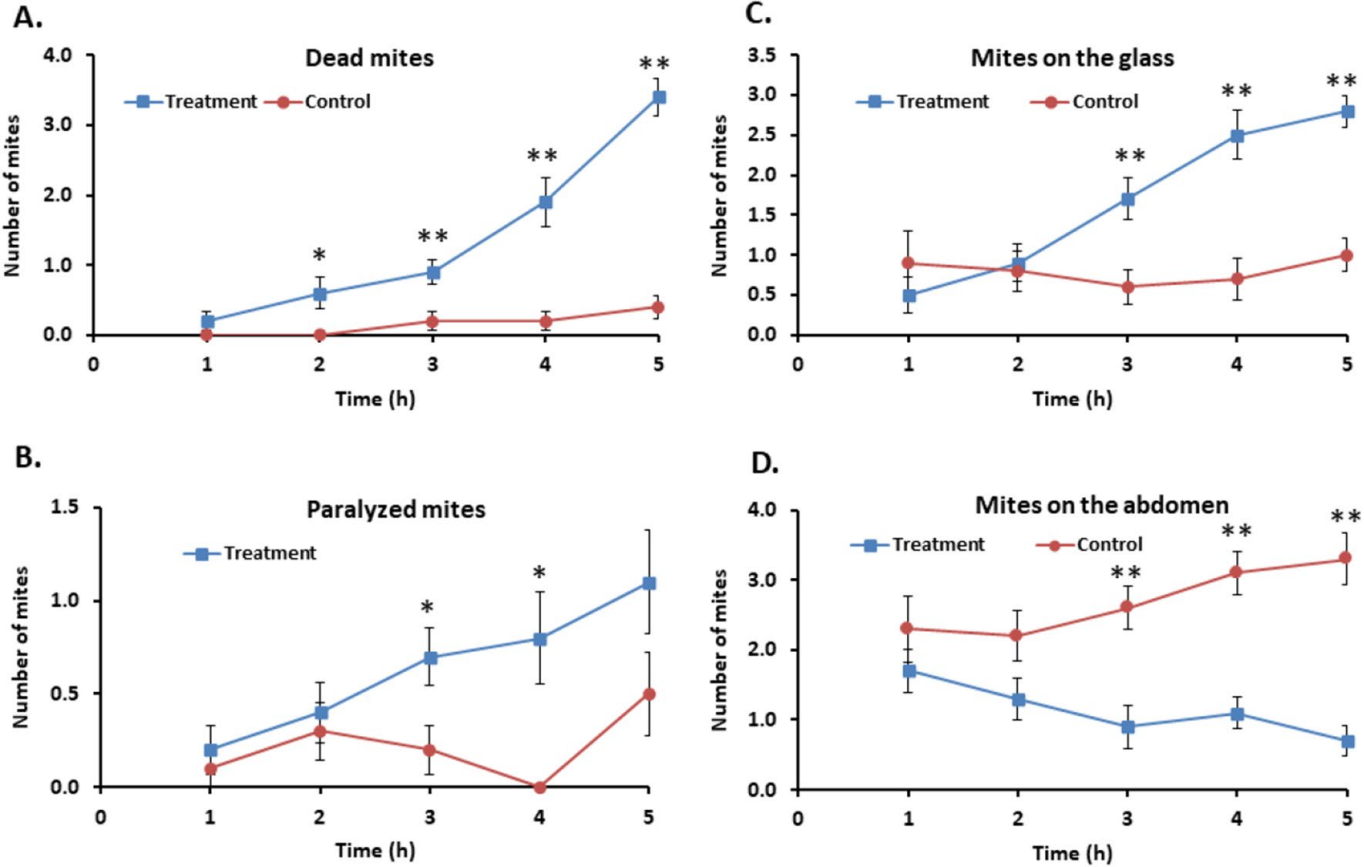
Time-course of mite death, paralysis and distribution, in the presence of compound **3c**{*3,6*} at a constant dose of 1 mg/replicate (Treatment) or no compound (Control). Each replicate had 5 mites and two fresh freeze-killed nurse bees as food in a glass Petri dish. See “Methods” for compound delivery and incubation details. Data points are average ± S. E. (10 replicates). (**A**) Number of dead mites vs. time. (**B**) Number of paralyzed mites vs. time. (**C**) Number of mites found on the glass. (**D**) Number of mites found on the abdomen of either of the bees. *P < 0.05 and **P < 0.01 (paired *t*-test between treatment and control at one time point).

Results for the time course are shown in Fig. 4, for mite death (A), mite paralysis (B), number of mites on the glass (C) and number of mites on the abdomen of either bee provided as food (D).

At 1 h, the number of dead mites did not differ between **3c**{*3,6*} treatment and the control (*t*-test, *P* > 0.05). At 2, 3, 4 and 5 h the number of dead mites in the treatment was significantly higher than in the control (Fig. 4A). Comparing the time points within one treatment series, there was no significant change from 1 to 5 h in the control (*P* > 0.05), but there was a significant increase in the **3c**{*3,6*} treatment (*P* < 0.05).

The number of paralyzed mites only differed significantly between treatment and control at 3 and 4 h. Within each series, there was no significant change in paralysis levels for the control, but there was a significant increase in the number of paralyzed mites from 1 to 5 h in the treatment (Fig. 4B).

The mites that were on the glass (Fig. 4C) did not differ significantly at the 1- and 2-h time points, but did differ at 3 h, 4 h and 5 h (*P* < 0.05). Within each treatment, there was no significant change in the control, but there was a significant increase of mites on the glass in the treatment (*P* < 0.05). For mites on the abdomen, there were significantly fewer mites in the treatment than in the control (*P* < 0.05) (Fig. 4D). Within the control series, there was no significant change over the 5 h period, but for the treatments there was a significant decrease of mites on the bee abdomen from 1 to 5 h (*P* < 0.05).

#### Dose responses

**Figure 5:**
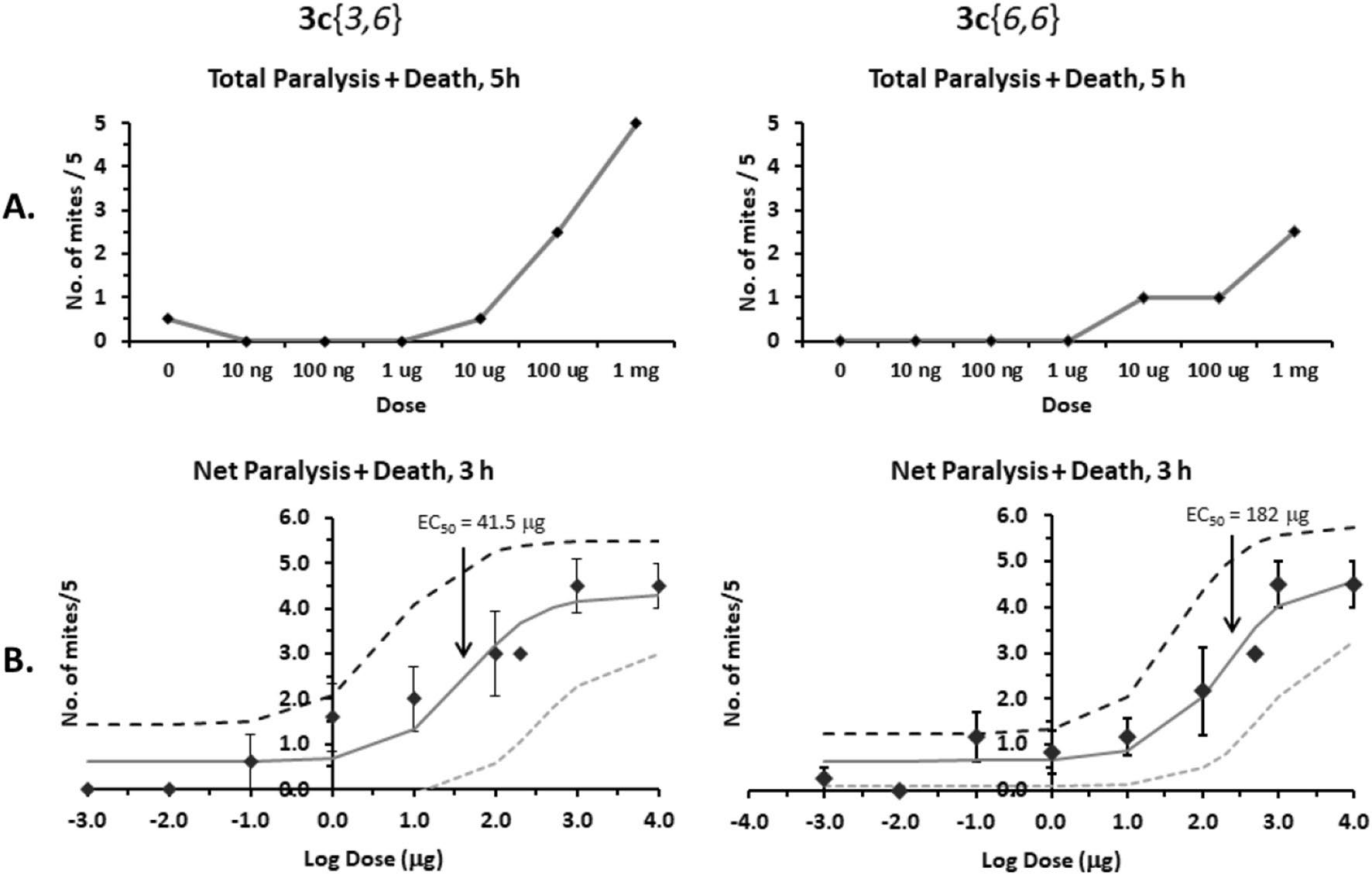
Dose responses of **3c**{*3,6*} and **3c**{*6,6*}. (**A**) Experiments done in AB. Points represent the total number of mites paralyzed and dead after 5 h of exposure, against the dose (average ± range, N = 2). (**B**) Experiments done in BC. Points represent the number of mites paralyzed and dead, minus the number of mites paralyzed and dead in the paired control without compound (see “Methods”), after 3 h of exposure. Points represent the average of 2–5 replicates per dose ± S.E. (for n ≥ 3) or range (for n = 2). The dark gray curve traces the calculated dose response, based on the EC_50_ and the activity range obtained. The light gray, stippled curve shows the low activity model, whereas the black, dashed curve shows the high activity model within the 95% confidence limits. The EC_50_ values obtained are indicated above the arrows.

Dose responses were obtained for compounds **3c**{*3,6*} and **3c**{*6,6*} (Fig. 5). All assays revealed that compound **3c**{*3,6*} is more active than **3c**{*6,6*}. After 5 h of exposure, mites were significantly paralyzed/killed by compound **3c**{*3,6*} at concentrations ≥ 10 µg/plate with half of the mites dead at 100 µg/plate and 100% mortality at 1 mg/plate (Odds Ratio = 4.26; 95% CI = 2.30–10.41; R^2^ = 0.492; Chi-square = 37.88; P < 0.0001), whereas compound **3c**{*6,6*} reached 50% mortality at 1 mg/plate (Odds Ratio = 3.14; 95% CI = 1.67–8.09; R^2^ = 0.338; Chi-square = 18.04; P < 0.0001) (Fig. 5A). After 3 h of exposure, the EC_50_ obtained for compound **3c**{*3,6*} was 41.5 μg/assay dish, whereas the EC_50_ for **3c**{*6,6*} was 182 μg/assay dish, or ca. 4 times less active than **3c**{*3,6*} (Fig. 5B). It is important to note that mites generally did not come in contact with the compound (unless they climbed onto the lid of the dish and onto the Parafilm, which was rare). Thus, the compound most likely exerts its effect through the gas phase.

#### Direct contact assays

**Figure 6:**
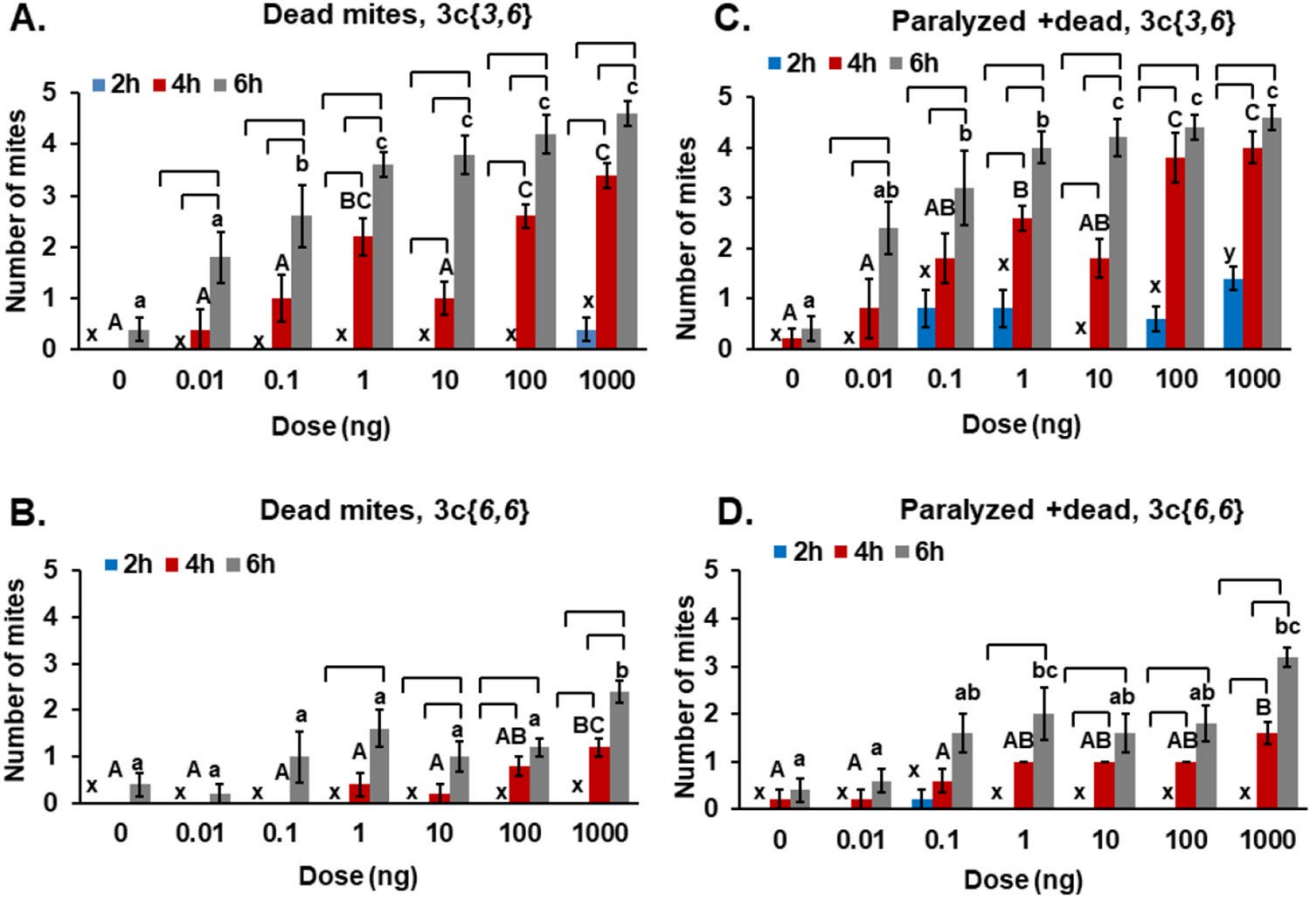
Direct contact assays of **3c**{*3,6*} and **3c**{*6,6*}. **A.** Number of dead mites (out of a total of five mites/replicate) treated with different doses of **3c**{*3,6*}, counted at 2 h (blue), 4 h (red) and 6 h (grey) after start of the experiment. **B.** Number of dead mites (out of a total of five mites/replicate) treated with different doses of **3c**{*6,6*}, counted at 2 h (blue), 4 h (red) and 6 h (grey) after start of the experiment. **C.** Total number of paralyzed and dead mites treated with different doses of **3c**{*3,6*}, counted at 2 h (blue), 4 h (red) and 6 h (grey) after start of the experiment. **D.** Total number of paralyzed and dead mites when treated with different doses of **3c**{*3,6*}, counted at 2 h (blue), 4 h (red) and 6 h (grey) after start of the experiment. A dose of 0 ng is the control treatment that received only ethanol. Bars represent mean ± S. E. of 5 replicates. Letters: comparison between each treatment dose and control at one time point- one-way ANOVA, followed by Tukey’s test, columns marked with different letters differ significantly p < 0.05: lower case xyz letters = 2 h, upper case ABC letters = 4 h and lower case abc letters = 6 h. Brackets above the letters indicate significant differences between the 2 h, 4 h and 6 h measurements for that dose—one-way ANOVA, followed by Tukey test (*P* < 0.05).

**Figure 7:**
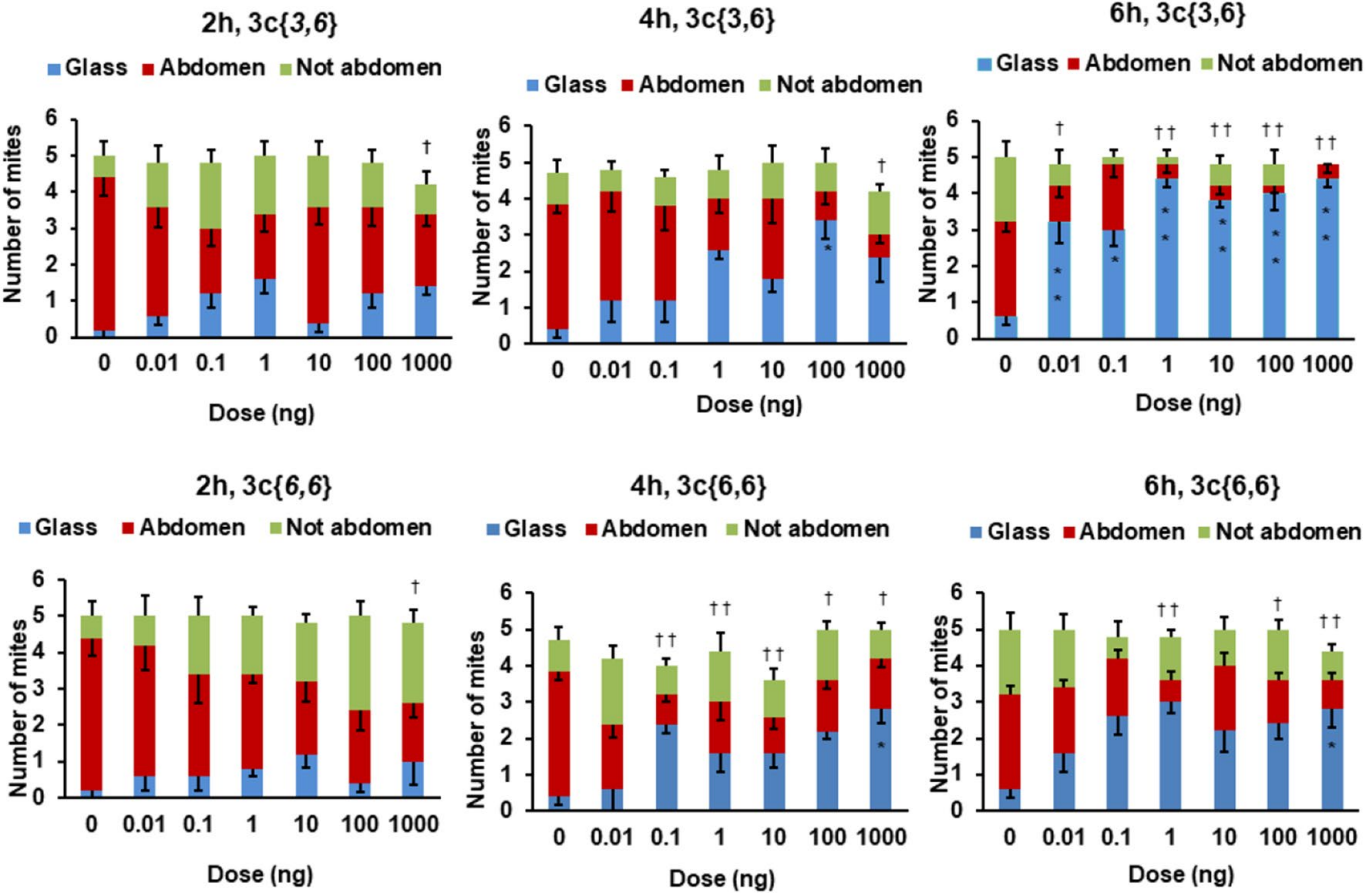
Distribution of mites in the direct contact assays. Mites found on the glass are shown in blue, mites found on the abdomen of either bee in the dish are shown in red and mites found on either bee but not on the abdomen are shown in green. Bars represent the mean ± S. E. of 5 replicates. Data analysed by comparing between each treatment dose for mite distribution (glass, abdomen or not abdomen) and control at one time point by one-way ANOVA, followed by Tukey’s test. Significant differences between the number of mites on the glass for a treatment dose to the control are indicated with a star (*P < 0.01; **P < 0.001). Significant differences between the numbers of mites on the abdomen for a treatment dose to the control are indicated with a dagger (^†^*P* < 0.01; ^††^*P* < 0.001).

Direct contact assays (Figs. 6 and 7) for acaricidal activity of **3c**{*3,6*} and **3c**{*6,6*} were analyzed for the number of dead mites, the total number of paralyzed and dead mites, the number of mites found on the glass and the number of mites found on the abdomen of any bees provided as food. Data are shown in Tables S2 and S3.

#### Mite death

At 2 h, there was no significant change in the numbers of dead mites between control and the different doses of **3c**{*3,6*}(Fig. 6A, blue bars). At 4 h, 1 ng, 100 ng and 1 µg of **3c**{*3,6*} had significantly higher numbers of dead mites than the control (Fig. 6A, red bars). At 6 h, all the doses of **3c**{*3,6*} had significantly more dead mites than the control or than a dose of 0.01 ng of **3c**{*3,6*} (Fig. 6A, grey bars). Comparing the time points within each dose, there were significant differences in the numbers of dead mites between 2 h, 4 h and 6 h exposures, starting at a dose of 0.01 ng. There was no significant increase in the number of dead mites for the control over the 6 h period.

At 2 h, the number of dead mites for any dose of **3c**{*6,6*} did not differ from the control (Fig. 6B). At 4 h, 100 ng and 1 µg of **3c**{*6,6*} had significantly more dead mites than the control (Fig. 6B, red bars). At 6 h, only a dose of 1 µg of **3c**{*6,6*} showed a significantly greater number of dead mites than the control (Fig. 6B, grey bars). Comparing the time points within one dose treatment, showed that there was no significant change in the number of dead mites in the control, and 0.01 ng and 0.1 ng doses of **3c**{*6,6*} over the 6-h treatment. At 6 h, doses from 1 ng to 1 µg of **3c**{*6,6*} had more dead mites than 2 h, and 10 ng and 1 µg of **3c**{*6,6*} had higher number of dead mites at 6 h than at 4 h. The doses of 100 ng and 1 µg of **3c**{*6,6*} at 4 h had more dead mites than at 2 h treatment.

#### Mite paralysis + death

At 2 h, 1 µg of **3c**{*3,6*} had significantly greater numbers of paralyzed + dead (P + D) mites than the control (Fig. 6C, blue bars). At 4 h, all the doses of **3c**{*3,6*} showed significantly more P + D mites than the control and 0.01 ng of **3c**{*3,6*} (Fig. 6C, red bars). At 6 h, all the doses had significantly higher numbers of P + D mites than the control (Fig. 6C, grey bars). Comparison within one dose treatment at different time points suggest no significant change in the numbers of P + D mites in the control at 2 h, 4 h or 6 h treatment times. Each dose of **3c**{*3,6*} had more P + D mites at 6 h than 2 h. The doses from 0.01 ng to 10 ng of **3c**{*3,6*} had significantly higher numbers of P + D mites at 6 h than 4 h. Each dose of **3c**{*3,6*} from 1 ng to 1 µg had more P + D mites at 4 h than at 2 h. The EC_50_ for death + paralysis of mites after 6 h of exposure for **3c**{*3,6*} was 0.06 ng/mite.

The numbers of P + D mites did not differ between control and the different doses of **3c**{*6,6*} at 2 h (Fig. 6D, blue bars). At 4 h, doses from 1 ng to 1 µg of **3c**{*6,6*} had significantly greater P + D mites than the control. At 6 h, each dose of **3c**{*6,6*} showed a significant increase in the numbers of P + D mites, compared to the control and 0.01 ng of **3c**{*6,6*}. Comparison within one dose treatment at different time points suggest no significant difference in the numbers of P + D mites in the control and 0.01 ng of **3c**{*6,6*} at 2 h, 4 h or 6 h treatment times. At 6 h, doses from 1 ng to 1 µg of **3c**{*6,6*} had higher numbers of P + D mites than 2 h. The dose of only 1 µg of **3c**{*6,6*} had more P + D mites at 6 h than 4 h. The doses of 10 ng, 100 ng and 1 µg of **3c**{*6,6*} had significantly greater numbers of P + D mites at 4 h than at 2 h. The EC_50_ for paralysis + death of mites after 6 h of treatment was 0.47 ng/mite.

#### Efficacy

At 4 h treatment, 70% of the mites were killed when treated with a dose of 1 µg of **3c**{*3,6*} and only 20% of the mites were killed with a dose of 1 µg of **3c**{*6,6*}. Furthermore, at 6 h, **3c**{*3,6*} showed 90% mortality of mites and **3c**{*6,6*} had 50% mortality at a dose of 1 µg. Altogether, these results show that **3c**{*3,6*} has higher acaricidal activity than **3c**{*6,6*} even when the mites come in direct contact with the treatments. Similar to the above results from the structure activity assays and dose responses, **3c**{*3,6*} acted faster than **3c**{*6,6*} in paralysing and killing mites.

#### Mite location on the glass

At 2 h, 1 ng and at 4 h, 1 ng,100 ng and 1 µg of **3c**{*3,6*} had significantly more mites on the glass than the control (Fig. 7 blue sections of the bars). At 6 h, all the doses of **3c**{*3,6*} showed significantly more mites on the glass than the control (Fig. 7, blue sections).

At 2 h, all the doses of **3c**{*6,6*} and the control showed no significant change in the number of mites on the glass (Fig. 7, blue sections). At 4 h, 0.1 ng, 100 ng and 1 µg of **3c**{*6,6*} had more mites on the glass than the control (Fig. 7, blue sections). At 6 h, 1 ng and 1 µg of **3c**{*6,6*} had significantly more mites on the glass than the control (Fig. 7, blue sections).

#### Mite location on the bee abdomen

At 2 h, 0.1 ng, 1 ng and 1 µg of **3c**{*3,6*} had significantly fewer number of mites on the bee abdomen than the control (Fig. 7, red sections). At 4 h, 100 ng and 1 µg treatments of **3c**{*3,6*} showed significantly fewer mites on the abdomen of the bees than the control (Fig. 7, red sections). At 6 h, all the doses of **3c**{*3,6*} showed significantly fewer mites on the bee abdomen than the control or 0.1 ng of **3c**{*3,6*}(Fig. 7, red sections).

At 2 h, the number of mites on the abdomen of the bees did not differ significantly in all the treatment doses of **3c**{*6,6*} and the control (Fig. 7, red sections). At 4 h, all the doses of **3c**{*6,6*} had significantly fewer mites on the bee abdomen than the control (Fig. 7, red sections). At 6 h, 1 ng, 100 ng and 1 µg of **3c**{*6,6*} had significantly lower number of mites on the bee abdomen than the control (Fig. 7, red sections).

#### Assays with blends

Because compounds **3c**{*3,6*} and **3c**{*6,6*} both had shown acaricidal activity (Fig. 2), we tested whether mixing the two compounds leads to synergism. Also, because **3b**{*2,2*} had shown host choice alteration activity previously^41^, we also checked if it is able to synergize with **3c**{*3,6*}. The results are shown in the Supplemental information (Figs. S5–S7). These assays showed that there is no synergism between the compounds when they are mixed.

### Acetylcholinesterase assays

#### Human AChE

A significant change in kinetics was only observed with DEET and **3b**{*2,2*}. At 3 mM of DEET, *K*_M_ was significantly increased, with no significant change in *V*_max_ (Table 1). Compound **3b**{*2,2*} showed a significant increase of *V*_max_ and *k*_cat_ at 3 mM. Lower concentrations of DEET and **3b**{*2,2*}, as well as the tested concentrations of **3c**{*3,6*}, and **3c**{*6,6*} did not have a significant effect on *V*_max_, *K*_M_, or *k*_cat_ of *h*AChE. Michaelis–Menten and Lineweaver–Burk plots for these data are shown in Fig. S9.

**Table 1:** Michaelis–Menten parameters of recombinant human acetylcholinesterase, *h*AChE, in the presence of compounds active against varroa.

Test compound	*V*_max_ (× 10^–5^ M/min)	*K*_M_ (× 10^–4^ M)	*k*_cat_ (× 10^3^ min^−1^)
None (control)	5.1 ± 0.2	1.0 ± 0.2	3.4 ± 0.2
0.5 mM DEET	6.5 ± 0.4	3.0 ± 0.4	4.4 ± 0.3
1 mM DEET	6.6 ± 0.7	4.0 ± 0.9	4.5 ± 0.5
3 mM DEET	7.9 ± 1.7	13.0 ± 4.0*	5.3 ± 1.1
0.5 mM **3c**{*3,6*}	4.4 ± 0.3	2.0 ± 0.3	3.0 ± 0.2
1 mM **3c**{*3,6*}	6.8 ± 0.6	4.0 ± 0.7	4.6 ± 0.4
3 mM **3c**{*3,6*}	5.5 ± 1.0	3.0 ± 1.0	3.7 ± 0.7
0.5 mM **3c**{*6,6*}	2.8 ± 0.2	1.0 ± 0.3	1.9 ± 0.1
1 mM **3c**{*6,6*}	5.3 ± 0.4	2.0 ± 0.4	3.6 ± 0.3
3 mM **3c**{*6,6*}	3.4 ± 0.5	0.7 ± 0.4	2.3 ± 0.3
0.5 mM **3b**{*2,2*}	2.8 ± 0.7	0.6 ± 0.9	1.9 ± 0.5
1 mM **3b**{*2,2*}	3.6 ± 0.3	0.8 ± 0.2	2.7 ± 0.2
3 mM **3b**{*2,2*}	8.2 ± 1.2*	5.0 ± 2.0	5.6 ± 0.8*

Values were obtained using GraphPad Prism 5 software analysis of rates obtained at different substrate concentrations using the Ellman assay (see text). Values shown are the kinetic constants obtained by least-squares fitting ± standard error. *k*_cat_ was calculated by dividing *V*_max_ by the concentration of AChE present in each 1 mL reaction (1.48 × 10^–8^ M).

*Significant difference compared to all other treatment groups (Tukey, *P* < 0.05).

#### Honey bee AChE

All three concentrations of **3c**{*3,6*} caused a significant decrease in *V*_max_ and specific activity, while 3 mM of **3b**{*2,2*}caused an increase in *V*_max_ and specific activity (Table 2). None of the tested compounds had a statistically discernible effect on *K*_M_ of *Am*AChE, though 3.0 mM DEET did show a higher average value for *K*_M_, similar to the other forms of AChE tested here. Michaelis–Menten and Lineweaver–Burk plots for these data are shown in Fig. S10.

**Table 2:** Michaelis–Menten parameters of extracted honey bee *Apis meliffera* acetylcholinesterase, *Am*AChE, in the presence of compounds active against varroa.

Test compound	*V*_max_ (× 10^–6^ M/min)	*K*_M_ (× 10^–5^ M)	Specific activity (μM/min μg)
None (control)	9.8 ± 0.4	2.2 ± 0.5	0.44 ± 0.02
0.5 mM DEET	10.9 ± 0.4	2.9 ± 0.5	0.49 ± 0.02
1 mM DEET	11.0 ± 0.3	4.4 ± 0.5	0.49 ± 0.01
3 mM DEET	9.7 ± 0.6	9.1 ± 1.8	0.44 ± 0.79
0.5 mM **3c**{*3,6*}	7.8 ± 0.3*	2.0 ± 0.4	0.35 ± 0.01*
1 mM **3c**{*3,6*}	6.7 ± 0.4***	2.9 ± 0.8	0.30 ± 0.02***
3 mM **3c**{*3,6*}	4.5 ± 0.2 ***	0.8 ± 0.3	0.20 ± 0.01***
0.5 mM **3c**{*6,6*}	10.5 ± 0.3	1.7 ± 0.2	0.47 ± 0.01
1 mM **3c**{*6,6*}	10.6 ± 0.2	1.4 ± 0.2	0.47 ± 0.01
3 mM **3c**{*6,6*}	10.0 ± 0.3	1.9 ± 0.3	0.45 ± 0.01
0.5 mM **3b**{*2,2*}	10.6 ± 0.2	1.5 ± 0.2	0.48 ± 0.01
1 mM **3b**{*2,2*}	10.6 ± 0.2	1.3 ± 0.2	0.47 ± 0.01
3 mM **3b**{*2,2*}	12.9 ± 1.2***	2.7 ± 1.3	0.58 ± 0.05***

Values were obtained using GraphPad Prism 5 software analysis of rates obtained at different substrate concentrations using the Ellman assay (see text). Values shown are the kinetic constants obtained by least-squares fitting ± standard error. Specific activity was calculated by dividing *V*_max_ by the concentration of protein isolate present in each 1 mL reaction (22.25 μg). * Significant difference compared to all other treatment groups (Tukey, *P* < 0.05), *** (Tukey, *P* < 0.001).

#### Varroa mite AChE

With *Vd*AChE, *V*_max_ values did not differ from the control in the presence of the various compounds (Table 3), except with 0.5 mM DEET (*V*_max_ and the specific activity were higher than the control) and 3.0 mM **3c**{*6,6*} (*V*_max_ and the specific activity were lower than the control). Only DEET at 3.0 mM showed a significant increase in *K*_M_. Compound **3c**{*3,6*} at any of the three concentrations tested had no effect on either *V*_max_ or *K*_M_. Michaelis–Menten and Lineweaver–Burk plots for these data are shown in Fig. S11.

**Table 3:** Michaelis–Menten parameters of extracted *Varroa destructor* acetylcholinesterase, *Vd*AChE, in the presence of compounds tested against Varroa mites.

Test compound	V_max_ (× 10^−6^ M/min)	K_M_ (× 10^−5^ M)	Specific activity (μM/min μg)
None (control)	1.1 ± 0.1	1.4 ± 0.6	0.16 ± 0.01
0.5 mM DEET	1.4 ± 0.1***	1.2 ± 0.4	0.20 ± 0.01***
1.0 mM DEET	1.2 ± 0.1	1.9 ± 0.6	0.17 ± 0.01
3.0 mM DEET	0.9 ± 0.1	5.4 ± 1.6***	0.13 ± 0.01
0.5 mM **3c**{*3,6*}	1.2 ± 0.1	1.2 ± 0.4	0.17 ± 0.01
1.0 mM **3c**{*3,6*}	1.3 ± 0.1	1.1 ± 0.3	0.19 ± 0.01
3.0 mM **3c**{*3,6*}	1.2 ± 0.1	1.2 ± 0.5	0.17 ± 0.01
3.0 mM **3c**{*6,6*}	0.8 ± 0.1**	1.4 ± 0.5	0.12 ± 0.01**
3.0 mM **3b**{*2,2*}	1.3 ± 0.1	1.4 ± 0.3	0.19 ± 0.01

Values were obtained using GraphPad Prism5 software. Rates were obtained at different substrate concentrations using the Ellman assay. Specific activity was calculated by dividing *V*_max_ by the weight of protein isolate present in each 1 ml reaction (6.86 μg).

**Significant difference compared to all other treatment groups (*P* < 0.01), *** (*P* < 0.001).

### Field trials

Before treatment application on Day 0 of the field trials, phoretic mite levels on adult bees did not differ between control and treatment colonies in BC (1.6 ± 0.8 vs 1.6 ± 1.1; *t*_18_ = -0.016, *P* = NS) or AB (11.5 ± 9.9 vs 11.6 ± 7.6; *t*_18_ = -0.021, *P* = NS) (Table 4). Nevertheless, at end of the experimental treatment period (Day 28), more mites were detected on bees from control than treatment colonies both in BC (3.5 ± 2.1 vs 1.3 ± 1.3; *t*_18_ = 2.8, *P* = 0.011) and AB (19.9 ± 13.6 vs 3.6 ± 2.5; *t*_18_ = 3.7, *P* = 0.041). On the final day of Apivar^®^ removal, similar numbers of mites on adult bees were found in the control and treatment colonies in BC (1.7 ± 2.9 vs 0.5 ± 0.7; *t*_18_ = 1.3, *P* = NS), whereas in AB, slightly greater numbers of mites per bee were found in the untreated control colonies (0.8 ± 0.7 vs 0.0 ± 0.0; *t*_18_ = 85, *P* = 0.0022).

**Table 4:** Data from the field trials of compound **3c**{*3,6*} in BC and AB.

Location	Experimental duration (2019)	Treatment	Alcohol washes (%)			Open brood (cm^2^)		Sealed brood (cm^2^)		Pollen (frame sides)	Honey (frame sides)	Brood cells with mites (%)
			Day 0 (treatment application)	Day 28 (treatment removal)	Final Day (Apivar^®^ removal)	Day 0	Day 28	Day 0	Day 28	Day 0	Day 0	Day 28
BC	Aug 7 –Oct 16	Control	1.6 ± 0.8	3.5 ± 2.1	1.7 ± 2.9	1379 ± 436	692 ± 539	2587 ± 317	1819 ± 290	0.7 ± 0.9	4.0 ± 0.8	18.7 ± 8.6
		**3c**{*3,6*}	1.6 ± 1.1	1.3 ± 1.3*	0.5 ± 0.7	1308 ± 421	348 ± 396	3375 ± 338	1066 ± 341	0.2 ± 0.4	3.4 ± 1.6	3.7 ± 3.7***^a^
AB	Aug 27–Nov 6	Control	11.5 ± 9.9	19.9 ± 13.6	0.8 ± 0.7	1274 ± 150	247 ± 80	1683 ± 158	671 ± 158	1.6 ± 0.6	2.3 ± 0.8	39.2 ± 21.3^*b*^
		**3c**{*3,6*}	11.6 ± 7.6	3.6 ± 2.5**	0.0 ± 0.0**	1235 ± 143	121 ± 58	1470 ± 171	427 ± 180	1.8 ± 0.4	2.5 ± 0.8	7.8 ± 5.6**^c^

Means ± S.E. of 10 replicates, unless noted.

Entries followed by * differed significantly between the **3c**{*3,6*} treatment and control [*P < 0.05: **P < 0.01; ***P < 0.001; all comparisons by *t*-tests or Mann–Whitney U tests (pollen stores only)].

^a^n = 7; ^b^n = 9; ^c^n = 5.

On Day 28, following removal of experimental treatments, we also examined the numbers of varroa mites in sealed brood cells, which is a measure of mite reproduction. The percentage of brood cells containing pupae infested with mites was significantly higher in control compared with treatment colonies in BC (18.7 ± 8.6 vs 3.7 ± 3.7; *t*_15_ = 5.2, *P* = 0.00029) as well as AB (39.2 ± 21.3 vs 7.8 ± 5.6; *t*_12_ = 4.4, *P* = 0.0014) (Table 4). All mites detected were successfully reproducing, with no dead mites or foundresses without progeny. Areas of sealed and unsealed brood did not significantly differ between treatment and control colonies, within locations (see Table 4).

**Figure 8:**
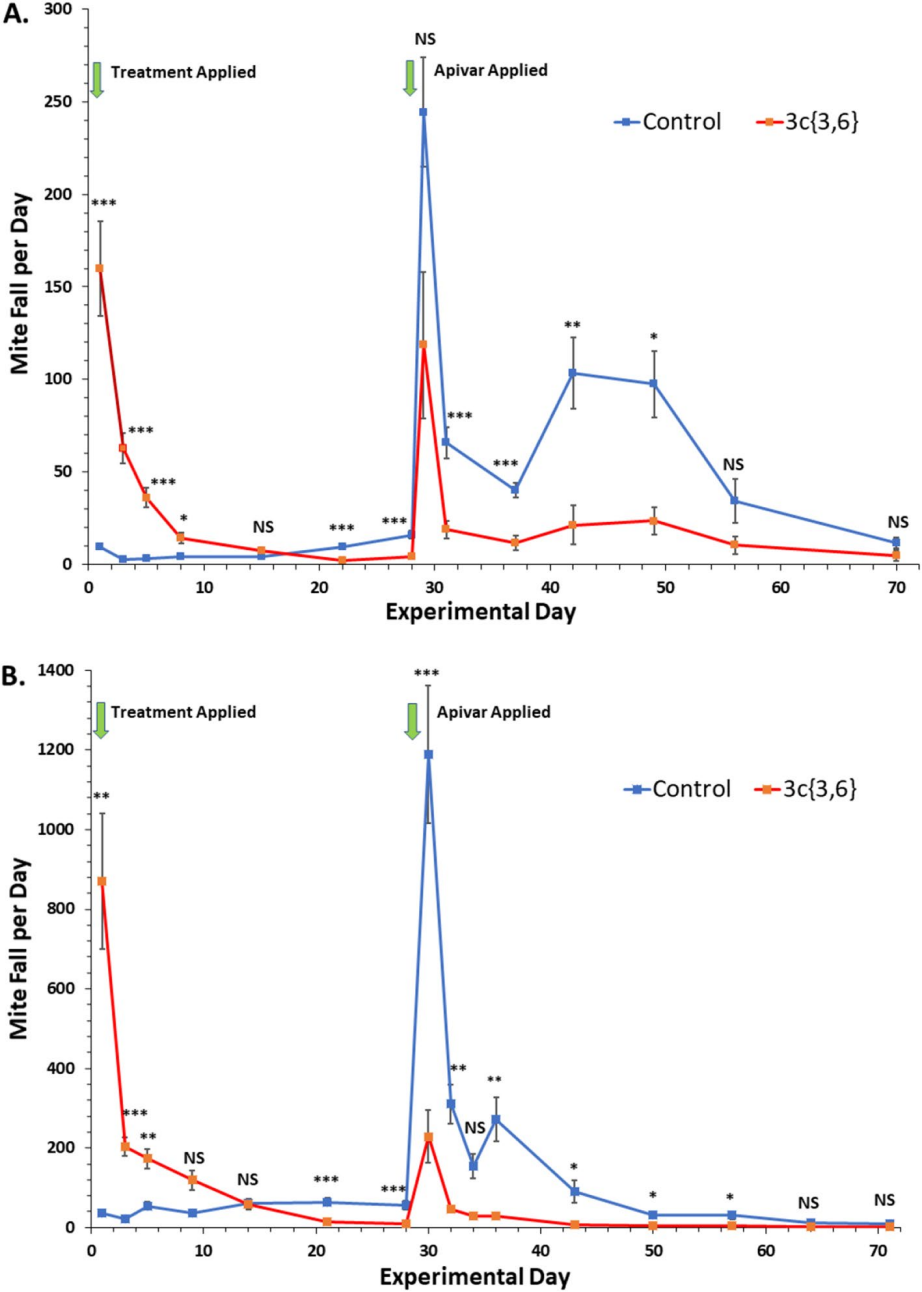
Time courses of the field trials of compound **3c**{*3,6*}, both performed in Canada. (**A**) Data from the trial in the lower Fraser Valley in British Columbia. (**B**) Data from the trial in Beaverlodge in Alberta. Points represent the average ± S. E. of 10 replicates. Blue: control devices, Orange: treatment devices containing 5 g of **3c**{*3,6*}. The experimental treatment lasted until the 28th day of the experiment, when the experimental devices were removed from the colonies and Apivar^®^ strips were installed for an additional 6 weeks. A generalized least squares model was fit to the untransformed results for mite fall. Pairwise comparisons between treatment and control were performed for each experimental day when sticky boards were counted from hives (*t*-tests, Bonferroni adjusted; *P < 0.05; **P < 0.01; ***P < 0.001; *NS* not significant).

Analysis of daily mite fall from sticky boards revealed consistent trends between our test apiaries located in two separate provinces and having distinctly different climates. For BC, our generalized least squares model showed significant main effects for both Treatment (F = 38.32; df = 1, 252; *P* = 0.0383) and Day (F = 7.18; df = 13, 252; *P* < 0.001), as well as the Treatment*Day interaction (F = 7.12; df = 13, 252; *P* < 0.001). In AB, similar significant main effects were also seen for Treatment (F = 12.68; df = 1, 288; *P* < 0.001), Day (F = 5.49; df = 15, 288; *P* < 0.001) and Treatment *Day (F = 5.52; df = 15, 288; *P* < 0.001). In BC, there was significantly higher mite fall on Days 1, 3, 5, and 8 after installation of the **3c**{*3,6*} treatment applicators compared with the controls, with very high rates of mite fall also seen in AB on Days 1, 3 and 5 (Fig. 8). By three weeks after treatment application, at both sites, the number of mites dropping to the bottom boards in the controls had risen above that of the treatments, indicating that the **3c**{*3,6*} was suppressing growth of the mite population. Reciprocal patterns for mite fall can be seen after the application of Apivar^®^ at each location, with significant differences on most sampling dates highlighting the greater density of mites remaining in colonies receiving the control treatments. In BC, despite the large spike in mite fall for the control caused by the introduction of Apivar^®^ as the finishing treatment on Day 28, no statistical difference in this measure was detected on Day 29 because of the large treatment variances and the moderate mite populations still present within the **3c**{*3,6*}-treated colonies. By day 56 in BC and Day 64 in AB, few mites remained in colonies with differences in daily mite fall no longer being detectable.

The overall efficacy of the compound in BC was calculated to be 51.2 ± 6.2%, whereas in AB it was 81.1 ± 2.9%, for a 4-week treatment (Table 5). Recovery of compound **3c**{*3,6*} from the applicators after their removal from the colonies demonstrated that ~ 1.2 g of compound remained on the combined applicators from each replicate. This meant that ~ 3.8 g evaporated during the 4-week treatment. In turn, this corresponds to an average daily evaporation of *ca.* 130 mg/day (Table 5). The monitoring devices (Porapak) installed in the colonies also indicated that the compound did evaporate throughout the treatment period. We detected the compound as follows: BC 1.0 ± 0.4 µg/device (week 1), 12.8 ± 1.8 µg/device (weeks 2–4) and for AB 0.24 ± 0.04 µg /device (week 1) with no compound detected from the devices covering weeks 2–4. In BC, 6.9 ± 2.9 µg/ ~ 1 g of compound was detected from beeswax, taken immediately after the experimental treatment, sampled near the location of the applicators, whereas in the samples from AB 0.5 ± 0.3 µg/ ~ 1 g was detected. In BC, 0.8 ± 0.3 µg/ ~ 1 g of compound was detected from a mixture of wax and honey sampled immediately after the experimental treatment from frames next to the applicators. In AB, no traces were detected in the honey/wax mixture taken after experimental treatment. It is noteworthy that a compound from wax, whose structure is to be determined, eluted at the same retention time as compound **3c**{*3,6*} in the GC–MS traces. Work is underway to optimize residue analysis of **3c**{*3,6*} in samples from hives.

**Table 5:** Overview of efficacy from the field trials of compound **3c**{*3,6*} in BC and AB.

Location	Treatment	Total mite fall per colony during treatment period	Total mite fall per colony during finishing period	Efficacy (%)^a^	Amount recovered from devices (mg)	Amount evaporated (mg)	Length of treatment (d)	Amount evaporated/day (mg)
BC	Control	231 ± 29	2221 ± 280	9.5 ± 0.6	N/A	N/A	28	N/A
	**3c**{*3,6*}	495 ± 63	636 ± 207	51.2 ± 6.2	1234 ± 158	3766 ± 158	28	134.5 ± 5.7
AB	Control	1527 ± 237	3876 ± 544	28.6 ± 2.3	N/A	N/A	28	N/A
	**3c**{*3,6*}	2576 ± 342	552 ± 88	81.1 ± 2.9	1041 ± 172	3959 ± 172	28	132.0 ± 5.7

Values represent means ± S.E. of 10 replicates per treatment.

^a^Efficacy calculated as: mite fall during the treatment period/(mite fall during treatment period + mite fall during finishing period) × 100.

In AB, control colonies suffered 100% mortality when evaluated the following spring (60% winter loss, 20% queenless, 20% very weak/functionally dead) while experimentally treated colonies had 40% colony mortality (30% winter loss, 10% very weak/functionally dead).

## Discussion

Here we describe the discovery of the acaricidal activity of 1-allyloxy-4-propoxybenzene, **3c**{*3,6*}, on *Varroa destructor*. Compound **3c**{*3,6*} had been shown previously to be the most active cabbage looper feeding deterrent from a large structure–activity survey^42^, as well as a mosquito repellent^43^. As such, it had already been tested for its physical properties^50^ and biodegradation by common environmental strains of the gram-negative soil bacterium *Pseudomonas putida*^51^. This compound was previously shown to decrease the olfactory responses of varroa mites to nurse bees and thereby to delay or even prevent host choice by varroa in choice bioassays^44^. In those assays we noticed that the delay or failure to complete a host choice was due to paralysis of the mites. This prompted us to study the compound and its structurally similar relatives further.

The active compounds described here displayed two different activities in the in vitro assays: (1) paralysis and eventual death of the mites (acaricidal activity) and (2) distribution of the mites away from the host bees (alteration of behavior). These two activities had similar, though not identical, structure–activity relationships (SAR) and time courses.

Acaricidal activity requires a *para* disposition of the two alkoxy groups on the central benzene ring. One of the two alkoxy groups needs to be an allyloxy group, whereas the other one can be: ethoxy (**3c**{*2,6*}), allyloxy (**3c**{*6,6*}), propoxy (**3c**{*3,6*}) or butoxy (**3c**{*4,6*}). The compound with the highest activity was **3c**{*3,6*}. Quantitative SAR revealed that the active compound needs to be within the size range of **3c**{*3,6*} (see above). The time course for **3c**{*3,6*} acaricidal activity (at ~ 50× the EC_50_) revealed that, in the non-contact assay (Fig. 4), two hours passed until a significant level of mite death or death + paralysis occurred. The time course for the contact assay (Fig. 6) at 17× the EC_50_ (1 ng applied) shows that four hours passed until a significant level of mite death + paralysis occurred. Thus, the acaricidal effect is not immediate. Similarly, the acaricidal effects of *tau*-fluvalinate, coumaphos and amitraz take ~ 6 h to manifest themselves^33,34^, as do the activities of hops extract (Hopguard^®^) and thymol^34^.

The alteration of behavior of the mites caused them to distribute on the glass of the assay dish instead of remaining attached to the bees that were provided for food. The time course of **3c**{*3,6*} activities showed that initially the mites did locate the bees and attached themselves, but after 3 h, they moved back onto the glass (Fig. 4). This observation is consistent with the host-seeking behavior of the mites, which is guided by odors emanating from hosts^52^. Mites walk in straight lines, making few turns, when no attractant is present. When an attractant (e.g. cuticular hydrocarbons, alcohols or aldehydes) is present, the mites make many turns to ensure that they stay arrested on the host^52^. It is also interesting to note that the mites on a bee were most often found on the abdomen in the controls or dishes with inactive compounds, consistent with other reports of preferences of phoretic mites for the abdomen of bees^6,7^. When exposed to the active compounds (thymol, **3c**{*3,6*}, **3c**{*6,6*}, **3c**{*3,3*} and **3a**{*3,6*}), the number of mites on the abdomen decreased, suggesting that these compounds interfere with arrestment of mites on a food source. How these compounds counter arrestment behavior of varroa is not known.

Compound **3c**{*3,6*} can evaporate from a hexane solution (with a liquid/gas equilibrium constant, K_l/g_, of 4.33 ± 0.12) or from solid surfaces to which it is adsorbed^50^. It has an octanol/water partition coefficient, logP, of 2.40 ± 0.17^50^. Furthermore, the compound can be fully biodegraded by common strains of *P. putida*, which are widespread in the environment. For example, *P. putida* ATCC 17453, which contains cytochrome P450_cam_ (CYP101A1) and its redox partners, first hydroxylates the CH_2_ group on the substituent chains next to the oxygen. This gives a hemiacetal, which loses the hydroxylated substituent group as an aldehyde. The resulting dihydroquinone was found to be completely degraded as well (*i.e.* it did not accumulate)^51^. Thus, compound **3c**{*3,6*} can evaporate, is of moderate hydrophobicity and can be biodegraded fully by common strains of bacteria. In future it will be interesting to determine whether bees, mites and/or hive microbiome members can also biodegrade this compound.

The volatility of compound **3c**{*3,6*} is important, as we noticed that the mites need not come in contact with the source of the compound during bioassays, for them to become paralyzed. Such a fumigant effect should be useful in a bee hive, as the compound could be released in the brood nest, evaporate and thereby diffuse to other parts of the colony. Another varroa acaricide that works through the gas phase is formic acid^53,54^.

Because compounds **3c**{*3,6*} and **3c**{*6,6*} caused paralysis in the mites, an activity that involves the central nervous system, we studied the effect of these compounds on acetylcholine esterase (AChE). Inhibition of AChE can cause paralysis, among other symptoms^55^, and it is important to ensure that neither human handlers nor honey bees will be negatively impacted by a varroa treatment. Also, as discovered in this and previous studies, a high concentration of DEET (≥ 3 mM) competitively inhibits AChE^47^. This inhibition manifests itself as an increase in *K*_M_ without a change in *V*_max_ or *k*_cat_. We found that all three forms of the enzyme, *h*AChE, *Am*AChE and *Vd*AChE, were competitively inhibited by DEET. Therefore, it was important to study the effect of the two paralysis-causing compounds (**3c**{*3,6*} and **3c**{*6,6*}) as well as the host choice-altering compound (**3b**{*2,2*}) on the three forms of AChE.

There was no significant increase in *K*_M_, or decrease in *V*_max_ or *k*_cat_ with **3c**{*3,6*}, and **3c**{*6,6*}on *h*AChE and *Vd*AChE, suggesting that they do not have an inhibitory effect on these enzymes. However, **3c**{*3,6*} did cause a decrease in *V*_max_ when tested on *Am*AChE. There are two forms of AChE in honey bees: one membrane-bound and one soluble. It is inconclusive from these studies what proportion of each was in the isolate we used for assays; therefore, it cannot be said whether one was more inhibited than the other. It does suggest that at least one AChE in honey bees is affected in an allosteric (*i.e.* non-competitive) mechanism, as has been seen in other invertebrates^56–58^. Bees exposed to high levels of **3c**{*3,6*} in their food for 1–2 weeks did not show higher mortality than controls^44^. Thus, the effect of **3c**{*3,6*} on *Am*AChE did not lead to acute symptoms. It remains to be seen whether there are behavioral or sublethal effects on bees. Compound **3c**{*3,6*} did not cause significant changes in *h*AChE. However, it should not be used in quantities that can accumulate to concentrations higher than 0.5 mM in acetyl cholinergic synapses. Such a high level of accumulation in an aqueous extracellular compartment is unlikely, given the moderate hydrophobicity and volatility of compound **3c**{*3,6*}^50^, as well as the low (μg) EC_50_ values we found in this study for varroa paralysis and death. The lack of any effect of **3c**{*3,6*} on *Vd*AChE indicates that this enzyme is not the target site of this compound in the mites. The mechanisms by which **3c**{*3,6*} paralyzes or alters arrestment behavior of mites still need to be discovered.

Compound **3b**{*2,2*}, showed a significant increase of *V*_max_ in both *h*AChE and *Am*AChE at the highest concentration of 3 mM; however, there was no significant change in *K*_M_. This suggests that **3b**{*2,2*} may have an allosteric effect on AChE which stimulates its activity^56,58^. *Vd*AChE was not affected by this compound. Although **3b**{*2,2*} did not cause inhibition of *h*AChE and *Am*AChE, and it altered the host choice of varroa mites^41^, it did not show acaricidal activity against varroa, so it will not be perused further.

The field trials of **3c**{*3,6*} emphatically demonstrated that this chemical causes mortality of phoretic mites in bee colonies, as measured by the numbers of mites dropping onto sticky boards. The efficacy of this compound in the field was 51.2 ± 6.2% in BC and 81.1 ± 2.9% in AB. These numbers are comparable to efficacies obtained by Gregorc et al.^34^ for Apiguard^®^ (thymol), Apistan^®^ (*tau*-fluvalinate), Apivar^®^ (amitraz) and Hopguard^®^ in similar experiments with 6-week experimental applications and 6-week follow-up treatments with coumaphos. In field trials during the fall of 2011 in Edmonton, AB, Vandervalk et al. (2014) tested Apivar^®^, formic acid, Hopguard^®^ and Thymovar^®^ in a 6-week application, with a follow-up treatment with oxalic acid after queens had stopped laying. They reported efficacies of 87.1 ± 2.7% (Apivar^®^), 78.5 ± 8.5 (formic acid), 88.9 ± 8.5 (Thymovar^®^), 43.0 ± 6.5% (Hopguard^®^) and 28.7 ± 7.3% (control)^59^. It is important to note that our trial only had a 4-week experimental treatment, rather than the 6 weeks more commonly used for other products. We also observed that the pattern of mite mortality at the two widely different geographic locations mirrored each other quite closely, even with the treatment periods being separated approximately 3 weeks in time. The lower efficacy of compound **3c**{*3,6*}in BC compared with AB is not likely due to the different starting infestation levels of mites in colonies: 1.6 ± 1.1% (BC) *vs*. 11.6 ± 7.6% (AB). We feel the difference in efficacy is most likely due to higher ambient fall temperatures in the Lower Mainland of BC which is conducive to the production of more late-season brood (Table 4), leading to greater mite reproduction thereby offsetting treatment-induced mite mortality. In both locations, **3c**{*3,6*}-treated colonies had significantly lower mite infestation levels at the end of the experimental treatment than the controls, and this pattern could also still be seen during and after the Apivar^®^ follow-up treatment. Similar patterns of infestation levels were observed by Gregorc et al.^34^ and Vandervalk et al.^59^ in their field trials of commercial acaricides.

In both provinces the number of mites found in sealed brood cells at the end of the experimental treatment was significantly lower in the treatment than in the controls. This phenomenon could be due to (1) the mites not arresting and entering the cells properly prior to cell capping, (2) fewer mites entering cells, or (3) a combination of (1) and (2). Given that mites failed to remain arrested on bees in the assays in dishes, it is plausible that the mites also failed to arrest on brood that was close to capping in the field trial. It is not known whether compound **3c**{*3,6*} can diffuse into sealed brood cells and/or affect mite reproduction, though we found no evidence of lack of mite fertility from **3c**{3,6} treatments in our field study.

In conclusion, compound **3c**{*3,6*} shows great promise in its ability to treat varroa infestations, by paralyzing the mites and causing them to eventually die. Additionally, the compound leads to mites not arresting on the abdomen of host bees, an activity that could synergize with paralysis and cause the dropping of mites onto the bottom board of a hive. Our field trial indicates that compound **3c**{*3,6*} is active in a hive environment and does not cause acute symptoms in bees, but it is still not known whether it accumulates in wax over many treatments or is able to vent or be biodegraded over periods that bees are not treated. Furthermore, its target site in the mites still needs to be discovered. Nevertheless, we believe that compound **3c**{*3,6*} is a promising lead in the search for new treatments against varroa infestations.

## Materials and methods

### Assays with mites

#### Bee and mite collection

Bees and mites for experiments performed in British Columbia (BC) came from three beekeepers in the Lower Mainland of BC in Burnaby (49.249° N 122.980° W), Port Moody (49.285° N 122.868° W) and Langley (49.104° N 122.660° W)). In Alberta (AB), bees and mites were collected from apiaries operated by Agriculture and Agri-Food Canada’s Beaverlodge Research Farm (55.203° N 119.396° W).

Freely moving varroa were collected, using a fine brush, either from the bottom board of a hive treated with powdered sugar, from a comb with emerging infested bees or from caged infested bees treated with CO_2_. Varroa were either used immediately or phoretically kept on young bees in cages until required. Forager bees were collected in front of the hive using a bee vacuum, and nurses observed to be feeding larvae were collected from a brood comb. For the bioassays, bees were frozen (−75 °C) and thawed just prior to experiments.

#### Bioassays in glass dishes with the compound evaporating from a source in the lid

To test host choice, paralysis or death of mites, assays were performed in glass Petri dishes (inner ∅ 9 cm), similarly to those previously described^41^. It is of note that plastic dishes do not work for these assays, because mites acquire static charge, and this interferes with the assay. Each dish had a ~ 2 × 2 cm square of Parafilm^®^, attached to the inner side of the lid, in the middle. Compounds were dispensed onto the Parafilm^®^ in 10 μL of hexane, using a glass syringe (Hamilton, Nevada). Control treatments received only solvent. The solvent was left to evaporate, then the lids were closed, and dishes were equilibrated at room temperature for 5–15 min. Next, one freshly thawed nurse and one forager were placed ~ 5 cm apart, equidistant from the center of the dish, and one live mite was taken from a holding dish (where mites were kept without food for 30 min) and placed in the middle of the dish. The dishes were placed in an incubator at 32–33 °C and ~ 40% to 70% humidity. In 2018, each replicate consisted of 5 dishes prepared in this way and each treatment replicate had a paired set of control dishes. Separate incubators were used for controls and treatments. Mite observations were carried out at various intervals ranging from 1 to 24 h (refer to “Results”). Net numbers of mites (see “Results”, Fig. S6 and Fig. 5B) were calculated by subtracting the numbers of dead or paralyzed mites in the paired control from those in the treatment.

In 2018, mites were observed with regard to whether they had made a host choice or not and their ability to move when touched with a fine brush. Mites that were paralyzed either moved slowly or twitched and moved their legs unproductively. Mites that did not move or twitch when touched were scored as dead. Occasionally, a mite could not be located and was scored as “lost”. Assays done in 2018 with this method were: (1) the initial screen of compounds **3c**{*2,6*}, **3c**{*3,3*}, **3c**{*3,4*}, **3c**{*4,6*}, **3c**{*3,6*} and **3c**{*6,6*}, (2) the dose responses of compounds **3c**{*3,6*} and **3c**{*6,6*}, and (3) experiments with mixtures of **3c**{*3,6*}, **3c**{*6,6*} and **3b**{*2,2*} (Fig. S8).

#### Structure–activity study in 2020

The same method of bioassay in the dishes as described above was used, except that each replicate consisted of five mites and two freshly freeze-killed nurse bees in one dish (i.e. no host choice was studied in these assays). The mites were placed in the middle and the bees on the periphery, as described above. The dose of the 15 compounds tested (Fig. 1) consisted of 5 μmol of compound in 10 μL of hexane (HPLC grade, distilled in glass), applied to the Parafilm^®^ square in the lid. The controls received 10 μL of distilled hexane. Six, nine or twelve replicates of each treatment were done. Dishes were set up and incubated as above and scored 3 h and 5 h after setup. Mites were scored for movement or death, as described above. Additionally, mites were scored for their location relative to the bees: on the glass, on the abdomen of the bee, or on a bee but not on the abdomen.

#### Time course in 2020

To study the timing of mite movement, paralysis and death in this assay system, 1 mg of **3c**{*3,6*} in 10 μL of hexane was applied to the Parafilm^®^ square. The same method as for the structure–activity study in 2020 was used. Each treatment was replicated ten times and scored for mite movement (paralysis or death) and position relative to the bees, 1, 2, 3, 4 and 5 h after setup.

#### Bioassays with direct application of compounds onto the mites

Five live mites were placed on a weighing paper (VWR), and each mite received 0.1 μL of solution of **3c**{*3,6*} or **3c**{*6,6*} in ethanol (HPLC grade) (treatments) or only ethanol (controls), using a 1 μL glass syringe (Hamilton, Nevada). A fresh weighing paper was used for each treatment. Once solvent had evaporated for 10–15 s, each treated mite was placed on the abdomen of a freshly freeze-killed bee, and this individual was placed in a glass Petri dish (inner ∅ 9 cm). Each replicate dish had five freeze-killed bees, each with one treated mite on its abdomen. Doses of **3c**{*3,6*} and **3c**{*6,6*} applied were: 10 ρg, 100 ρg, 1 ng, 10 ng, 100 ng and 1 μg. Five replicates of each dose of compound and of ethanol controls were tested. The experimental dishes were incubated at 32–33 °C and ~ 40% to 70% humidity. The mites were scored after 2, 3, 4, 5 and 6 h, for paralysis or death, and for location relative to the bees: on the glass, on the abdomen of the bee or on a bee but not on the abdomen.

### Acetylcholinesterase assays

#### Buffer and stock solutions

Sodium phosphate buffer, 100 mM, pH 7.6 was used to make a 1 mM stock solution of acetylthiocholine iodide (Sigma Aldrich) substrate, and a 10 mM stock of 5,5’-dithiobis-2-nitro-benzoic acid (DTNB), to produce a coloured anion for every acetylthiocholine hydrolyzed (Fig. S1). The DTNB stock also contained 18 mM NaHCO_3_.

We tested the effect of four compounds on AChE; DEET (Sigma-Aldrich, Oakville, Ontario, Canada), 1-allyloxy-4-propoxy benzene (**3c**{*3,6*}), 1,4-diallyloxy benzene (**3c**{*6,6*}), and 1,3-diethoxy benzene (**3b**{*2,2*}) (Fig. 1). Stock solutions of 25 mM, 50 mM, and 150 mM were dissolved in ethanol, and 55% Tween 20 (v/v) was added to prevent precipitation of inhibitors during runs (Supplementary Information).

Human expressed AChE (*h*AChE), (C1682) was obtained from Sigma-Aldrich Canada Co. (Oakville, Ontario). The lyophilized powder was dissolved in sterile phosphate buffer with 10% (v/v) sterile glycerol, yielding a concentration of 0.1 mg/ml. The *h*AChE was then aliquoted and flash-frozen before being stored at − 75 °C until assays were performed.

Honey bee AChE (*Am*AChE) was isolated from pre-frozen nurse bees collected from multiple colonies in BC, and the concentration of the isolate was determined using the Bradford assay (Supplementary Information). *Varroa destructor* AChE (*Vd*AChE) was isolated from pre-frozen mites collected from multiple colonies in BC as described for *Am*AChE (see Supplementary Information).

#### Ellman assays—human AChE

These assays were adapted from ones described previously^47–49^. For *h*AChE, we tested ten different substrate concentrations of acetylthiocholine iodide substrate between 870 and 8.7 μM, and 3 replicates were performed per substrate concentration. Substrate (870 μL of stock solutions, ranging from 10 μM to1.0 mM), DTNB (100 μL of 10 mM stock in buffer—see above), and the compound (20 μL stock in ethanol with Tween 20—see above) were added to the cuvette, the cuvette was inverted several times, and the solution was used to blank the spectrophotometer. Stock *h*AChE was added (10 μL to give 1.48 × 10^–6^ M), the cuvette was inverted once, and the run was started as soon as possible. Runs were performed at 426 nm; the wavelength experimentally determined to give the highest absorption of 5-thiolate-2-nitrobenzoate (supplementary information). Runs had duration of three minutes, and absorbance was measured every 5 s, yielding 36 data points per run.

An extinction coefficient of 11,792 cm^−1^ M^−1^ was used to convert absorbance to concentration (Supplementary Information). Concentration was plotted against time to determine the rate of the reaction. The first three data points were omitted due to variant mixing effects of AChE with the buffer, DTNB, and compound. The rate of product conversion was determined by examining the linear region of the kinetics curve, including as many data points as possible with an R^2^ ≥ 0.99. The rates for the three replicates were then averaged before plotting them against substrate concentration to determine *V*_max_, *K*_M_ and *k*_cat_ using Grafit5 software analysis (Version 5.0.6, Erithacus Software Limited, West Sussex, UK). Errors are fitting errors.

#### Honey bee AChE

The same experimental procedure was used for *Am*AChE as for the human form. However, since the isolated *Am*AChE was not as concentrated, we used twice as much volume of protein solution in the cuvette. Therefore, the volumes and concentrations of solutions added to the cuvette were: substrate (860 μL, final concentration 8.6 μM to 0.86 mM), DTNB (100 μL, final concentration 1 mM), inhibitor (20 μL, final concentration 0.5–3.0 mM), *Am*AChE isolate (20 μL, 445 ng protein).

#### Varroa AChE

For this assay, we tested ten different concentrations of acetylthiocholine iodide substrate from 10 μM to 1 mM, prepared from a 2 mM stock. DTNB stock (10 mM, 100 µL) was mixed in the cuvette with 20 µL of ethanol/ 55%Tween 20 (v/v) solution of the test compound (see above) or control. Substrate solutions were added to the cuvette, followed by phosphate buffer pH 7.6 to make a total of 1.0 mL of solution. The cuvette was inverted three times and used to blank the spectrophotometer. After addition of 60 µL of varroa extract, the cuvette was inverted once, and the run was started as quickly as possible. All the runs were taken at 420 nm for total 3 min. Absorbance was recorded every 5 s to yield 36 data points per run. Analysis was done by the same procedure as described for *h*AChE and *Am*AChE. Errors are fitting errors.

### Field trials

In 2019, we performed two field trials at different geographic locations: (1) the Fraser Valley (Langley), BC and (2) Beaverlodge, AB (at AAFC’s Beaverlodge Research Farm). Bee colonies were infested with varroa earlier in the summer, to create populations of mites that would nominally exceed treatment thresholds for Canada during the fall^53^. Colonies were standardized to six frames of bees, five frames of predominantly open brood and a 1-year-old locally-bred queen. Each trial consisted of ten single-brood chamber Langstroth colonies per treatment, with two treatments: no compound (control) and 5 g of compound/colony. The compound was delivered as a solid (83.3% w/w **3c** {*3,6*} and 16.7% w/w glycerol), applied to large wooden craft sticks (Fig. S2). The controls received the wooden sticks with only glycerol. Each stick contained 500 mg of **3c**{*3,6*}, applied evenly over both sides, and there were 10 sticks/hive. In each location there were ten colonies with **3c**{*3,6*} treatment and ten colonies with the control. Sticks were suspended between brood frames (Fig. S2). In BC, phoretic mite levels were assessed by alcohol washes of ~ 300 adult workers (isopropanol:water 1:1) in EasyCheck devices (Véto-pharma, Palaiseau, France), while in AB, mite levels were washed in 70% (v/v) ethanol using screened-bottom containers placed on a laboratory benchtop shaker table. Brood areas were measured as 2.54 × 2.54 cm squares by comparison to a wooden frame threaded with a grid of that size. Areas of food stores were measured to the nearest quarter of a single frame side.

Both trials had an initial experimental treatment period for the application of **3c**{*3,6*}, or control devices without any active compound. For BC, this phase of the trial started August 7, 2019 and ended September 4, 2019. The trial in AB started August 27, 2019 and ended September 25, 2019. Within each location, 20 colonies with similar quantities of open brood, capped brood, pollen stores and honey stores, and having similar phoretic mite infestations were used to start the experiment (Day 0) (Table 4). Each colony had a removable sticky board placed beneath a screened bottom board to collect falling mites, which were counted on experimental days 1, 3, 5, 8, 15, 22 and 28 (BC), or days 1, 3, 5, 9, 14, 21 and 28 (AB). On the last day of experimental treatment period (Day 28), the wooden sticks were removed and hives were assessed for phoretic mite levels, areas of brood and stores, general health, queen status and mite reproduction. The latter was done by choosing one comb of sealed brood from each colony and opening 100 cells containing purple-eyed pupae. Cells were uncapped and carefully examined under a dissecting microscope to verify the presence and number of foundresses, offspring, and any dead mites.

Following the experimental treatment period described above, all colonies were treated with Apivar^®^ strips (3.33% amitraz) as per manufacturer’s instructions (two strips/brood chamber; Véto-pharma, Palaiseau, France). This finishing period lasted 42 days in both locations. Monitoring of mite drop using sticky boards continued during the finishing period on experimental days 29, 31, 37, 42, 49, 56 and 70 (BC), or days 30, 32, 34, 36, 43, 50, 57, 64 and 71 (AB).

Apart from monitoring mite drop, the rate of fumigation by **3c**{*3,6*} was determined in two ways: (1) by using Pasteur pipettes filled with Porapak Q 80–100 mesh (Millipore, Oakville, Canada), a sorbent, and (2) by recovering the release devices and analyzing how much compound was left. Wax and honey samples were collected on the last day of experimental treatment. These were extracted and analyzed for **3c**{*3,6*} (see “Supplemental Methods” for the extraction and analysis method).

In AB, colonies were maintained as intact experimental groups for wintering. Following experimental activities, all colonies were fed sugar syrup (67 brix) and moved indoors to overwinter at 4 ± 1 °C on 7 November 2019. Colonies were moved out of the wintering building on 15 April 2020 and visually assessed on 21 April and 11 May 2020 for mortality. Those colonies not dead but weak (having less than one frame of bees) or queenless, were scored as being “functionally dead”.

### Statistical analyses

For AChE assays, GraphPad Prism5 was used to perform all statistical analysis (GraphPad software Inc., La Jolla, CA, USA). One-way ANOVA, followed by Tukey’s test was performed to determine significant difference (*P* < 0.05) for *V*_max_, *K*_M_, *k*_cat_, and specific activity between each concentration of inhibitor and the control.

For behavioral assays, GraphPad Prism5 was used to perform analysis. Data from the structure–activity assays were analyzed by ANOVA and pairwise comparison using either Kruskal–Wallis or Tukey’s tests. For the dose responses (Fig. 5B) data were fitted to the dose response function in GraphPad Prism (Eq. 1), where *E* is the activity, *E*_max_ is the maximal activity, *A* is the dose of the compound in μg and *EC*_50_ is the dose at which half-maximal activity is attained.

1 $$\frac{E}{Emax}=1/(1+(\frac{logEC50}{logA})$$

A binomial logistic regression model was used to assess whether compounds **3c**{*3,6*} and **3c**{*6,6*} killed/paralyzed Varroa after 5 h of exposure (Fig. 5A). These statistical analyses were carried out using SAS JMP^®^ 14.

For field experiments, comparisons between treatment means within sites for phoretic mite levels (% mites per adult bees from alcohol washes), as well as brood areas, areas of food stores and mite infestation densities (% sealed brood cells containing mites) were performed using two-sided, two-sample Student’s *t*-tests. In cases where sample sizes were unequal or standard error estimates for the treatments differed vastly, Welch’s *t*-test was applied. Mann–Whitney U tests were employed to compare areas of pollen stores because of the highly non-normal nature of the data.

Data for mite fall on sticky boards were first normalized into mite fall per day by dividing the mite count by the number of days the board resided in the colony. A generalized least-squares model was fit to the untransformed results for mite fall per day that included a two-level treatment factor (treatment vs control), a 14 level (for BC) or 16 level (for AB) categorical variable for experimental day, as well as their interactions as the predictor variables. Mite fall was analyzed separately for each location. To handle the range of variances for the large range of counts, a separate variance was estimated for each Treatment × Day (categorical) combination. Various correlation structures for the repeated measures on Colony were explored but were not successful at removing the very strong autocorrelation. A Bonferroni multiple testing adjustment was applied to the tests of differences between treatment *vs*. control as well as the 95% confidence intervals for the 14 or 16 comparisons at each experimental day. Analyses for field experiments were performed using R version 4.1.3 (2022-03-10)^60^ and RStudio version 2022.2.1.461 (2022-03-17)^61^.

## Supplementary Information

Supplementary Information.

## Data Availability

The datasets used and/or analysed during the current study are available upon request from the corresponding author.

## References

[1] Klein A-M (2007). Importance of pollinators in changing landscapes for world crops. Proc. R. Soc. Lond. Ser. B. Biol. Sci..

[2] Caldernoe NW (2012). Insect pollinated crops, insect pollinators and US Agriculture: Trend analysis of aggregate data for the period 1992–2009. PLoS ONE.

[3] Ratnieks FLW, Carreck NL (2010). Clarity on honey bee collapse?. Science.

[4] Steinhauer N (2018). Drivers of colony losses. Curr. Opin. Insect Sci..

[5] Genersch E (2010). Honey bee pathology: Current threats to honey bees and beekeeping. Appl. Microbiol. Biotechnol..

[6] Egekwu NI, Posada F, Sonenshine DE, Cook S (2018). Using an *in vitro* system for maintining *Varroa destructor* mites on *Apis** mellifera* as hosts: studies of mite longevity and feeding behavior. Exp. Appl. Acarol..

[7] Ramsey S (2019). *Varroa destructor* feeds primarily on honey bee fat body tissue and not hemolymph. Proc. Natl. Acad. Sci. USA.

[8] Beaurepaire A (2020). Diversity and global distribution of viruses of the Western honey bee, *Apis** mellifera*. Insects.

[9] Evans JD, Cook SC (2018). Genetics and physiology of varroa mites. Curr. Opin. Insect Sci..

[10] Rosenkranz P, Aumeier P, Ziegelmann B (2010). Biology and control of *Varroa destructor*. J. Invert. Pathol..

[11] Frey E, Rosenkranz P (2014). Autumn invasion rates of *Varroa destructor* (Mesostigmata: Varroidae) into honey bee (Hymenoptera: Apidae) colonies and the resulting increase in mite populations. J. Econ. Entomol..

[12] Guzman-Novoa E (2010). *Varroa destructor* is the main culprit for the death and reduced populations of overwintered honey bee (*Apis** mellifera*) colonies in Ontario, Canada. Apidologie.

[13] Haber AI, Steinhauer NA, van Engelsdorp D (2019). Use of chemical and nonchemical methods for the control of *Varroa destructor* (Acari: Varroidae) and associated winter colony losses in U.S. beekeeping operations. J. Econ. Entomol..

[14] DeGrandi-Hoffman G, Ahumada F, Probasco G, Schantz L (2012). The effects of beta acids from hops (*Humulus** lupulus*) on mortality of *Varroa destructor* (Acari: Varroidae). Exp. Appl. Acarol..

[15] Imdorf A, Bogdanov S, Ochoa RI, Calderone NW (1999). Use of essential oils for the control of *Varroa **jacobsoni* Oud. in honey bee colonies. Apidologie.

[16] Rademacher E, Harz M (2006). Oxalic acid for the control of varroosis in honey bee colonies—A review. Apidologie.

[17] Lindberg CM, Melathopoulos AP, Winston ML (2000). Laboratory evaluation of miticides to control *Varroa **jacobsoni* (Acari: Varroidae), a honey bee (Hymenoptera: Apidae) parasite. J. Econ. Entomol..

[18] Plettner E, Eliash N, Singh NK, Pinnelli GR, Soroker V (2017). The chemical ecology of host-parasite interaction as a target of *Varroa destructor* control agents. Apidologie.

[19] Bogdanov S, Imdorf A, Kilchenmann V (1998). Residues in wax and honey after Apilife VAR treatment. Apidologie.

[20] Chaimanee V, Johnson J, Pettis JS (2022). Determination of amitraz and its metabolites residue in honey and beeswax after Apivar® treatment in honey bee (*Apis** mellifera*) colonies. J. Apicult. Res..

[21] Medici SK, Castro A, Sarlo EG, Marioli JM, Eguaras MJ (2012). The concentration effect of selected acaricides present in beeswax foundation on the survival of *Apis** mellifera* colonies. J. Apicult. Res..

[22] Payne AH, Walsh EM, Rangel J (2019). Initial exposure of wax foundation to agrochemicals causes negligible effects on the growth and winter survival of incipient honey bee (*Apis** mellifera*) colonies. Insects.

[23] Hubert J (2014). Point mutations in the sodium channel gene conferring tau-fluvalinate resistance in *Varroa destructor*. Pest. Manag. Sci..

[24] Kamler M, Newvorna M, Stara J, Erban T, Hubert J (2016). Comparison of *tau*-fluvalinate, acrinathrin and amitraz effects on susceptible and resistant populations of *Varroa destructor* in a vial test. Exp. Appl. Acarol..

[25] Maggi MD, Ruffinengo SR, Gende LB, Eguaras MJ, Sardella NH (2008). LC_50_ baseline levels of amitraz, coumaphos, fluvalinate and flumethrin in populations of *Varroa destructor* from Buenos Aires Province, Argentina. J. Apicult. Res..

[26] Millán-Leiva A, Marín O, Christmon K, van Engelsdorp D, González-Cabrera J (2021). Mutations associated with pyrethroid resistance in Varroa mite, a parasite of honey bees, are widespread across the United States. Pest. Manag. Sci..

[27] Millán-Leiva A (2021). Mutations associated with pyrethroid resistance in the honey bee parasite *Varroa destructor* evolved as a series of parallel and sequential events. J. Pest. Sci..

[28] Pettis J (2004). A Scientific note on *Varroa destructor* resistance to coumaphos in the United States. Apiologie.

[29] Vlogiannitis S (2021). Reduced proinsecticide activation by cytochrome P450 confers coumaphos resistance in the major bee parasite *Varroa destructor*. Proc. Natl. Acad. Sci. USA.

[30] Abou-Donia MB, Dechkovskaia AM, Goldstein LB, Abdel-Rahman A, Bullman SL, Khan WA (2004). Co-exposure to pyridostigmine bromide, DEET, and/or permethrin causes sensorimotor deficit and alterations in brain acetylcholinesterase activity. Pharmacol. Biochem. Behav..

[31] Boily M, Sarrasin B, DeBlois C, Aras P, Chagnon M (2013). Acetylcholinesterase in honey bees (*Apis** mellifera*) exposed to neonicotinoids, atrazine and glyphosate: Laboratory and field experiments. Env. Sci. Pollution Res..

[32] Vu PD, Rault LC, Jenson LJ, Bloomquist JR, Anderson TD (2020). Voltage-gated chloride channel blocker DIDS as an acaricide for *Varroa* mites. Pestic. Biochem. Phys..

[33] Roth MA, Wilson JM, Gross AD (2021). Assessing *Varroa destructor* acaricide resistance in *Apis** mellifera* colonies of Virginia. Apidologie.

[34] Gregorc A, Alburaki M, Sampson B, Knight PR, Adamczyk J (2018). Toxicity of selected Acaricides to honey bees (*Apis** mellifera*) and Varroa (*Varroa destructor* Andersen and Trueman) and their use in controlling varroa within honey bee colonies. Insects.

[35] Rinkevich FD (2020). Detection of amitraz resistance and reduced treatment efficacy in the Varroa Mite, *Varroa destructor,* within commercial beekeeping operations. PLoS ONE.

[36] González-Cabrera J (2018). A single mutation is driving resistance to pyrethroids in European populations of the parasitic mite, *Varroa destructor*. J. Pest. Sci..

[37] Milani N, Della Vedova G (2002). Decline in the proportion of mites resistant to fluvalinate in a population of *Varroa destructor* not treated with pyrethroids. Apidologie.

[38] Riva C (2019). In silico chemical library screening and experimental validation of novel compounds with potential varroacide activities. Pestic. Biochem. Phys..

[39] Bahreini R (2020). Evaluation of potential miticide toxicity to *Varroa destructor* and honey bees, *Apis** mellifera*, under laboratory conditions. Sci. Rep..

[40] Bahreini R (2022). Miticidal activity of fenazaquin and fenpyroximate against *Varroa*
*destructor*, anectoparasite of *Apis*
*mellifer*a. Pest. Manag. Sci..

[41] Eliash N (2014). Can we disrupt the sensing of honey bees by the bee parasite *Varroa*
*destructor*?. PLoS ONE.

[42] Akhtar Y, Yu Y, Plettner E, Isman MB (2010). Dialkoxybenzene and dialkoxy-allylbenzene feeding and oviposition deterrents against the cabbage looper, *Trichoplusia*
*ni*: Potential insect behavior control agents. J. Agric. Food Chem..

[43] Hodson CN, Yu Y, Plettner E, Roitberg BD (2016). New repellent effective against African malaria mosquito *Anopheles*
*gambiae*: Implications for vector control. Med. Vet. Entomol..

[44] Singh NK (2020). Effect of the insect feeding deterrent 1-allyloxy-4-propoxybenzene on olfactory responses and host choice of *Varroa*
*destructor*. Apidologie.

[45] Singh NK (2015). The effect of DEET on chemosensing of the honey bee and its parasite *Varroa*
*destructor*. Apidologie.

[46] Colovic MB, Krstic DZ, Lazarevic-Pasti TD, Bondzic AM, Vasic VM (2013). Acetylcholinesterase inhibitors: Pharmacology and toxicology. Curr. Neuropharmacol..

[47] Corbel V (2009). Evidence for inhibition of cholinesterases in insect and mammalian nervous systems by the insect repellent deet. BMC Biol..

[48] Ellman GL, Courtne YKD, Andres VJ, Featherstone RM (1961). A new and rapid colorimetric determination of acetylcholinesterase activity. Biochem. Pharmacol..

[49] Worek F, Eyer P, Thiermann H (2011). Determination of acetylcholinesterase activity by the Ellman assay: A versatile tool for in vitro research on medical countermeasures against organophosphate poisoning. Drug Test. Anal..

[50] Ebrahimi P, Spooner J, Weinberg N, Plettner E (2013). Partition, sorption and structure activity relation study of dialkoxybenzenes that modulate insect behavior. Chemosphere.

[51] Ebrahimi P, Plettner E (2014). Biodegradation of 1-allyloxy-4-propoxybenzene by selected strains of *Pseudomonas*
*putida*. Biodegradation.

[52] Donzé G (1998). Aliphatic alcohols and aldehydes of the honey bee cocoon induce arrestment behavior in *Varroa*
*jacobsoni* (Acari: Mesotigmata), an ectoparasite of *Apis*
*mellifera*. Arch. Insect Biochem. Physiol..

[53] Currie RW, Gatien P (2005). Timing acaricide treatments to prevent *Varroa*
*destructor* (Acari: Varroidae) from causing economic damage to honey bee colonies. Can. Entomol..

[54] Ostermann DJ, Currie RW (2004). Effect of formic acid formulations on honey bee (Hymenoptera: Apidae) colonies and influence of colony and ambient conditions on formic acid concentration in the hive. J. Econ. Entomol..

[55] Eddleston M, Buckly NA, Eyer P, Dawson AH (2008). Management of acute organophosphate pesticide poisoning. Lancet.

[56] Marcel V, Palacios LG, Pertuy C, Masson P, Fournier D (1998). Two invertebrate acetylcholinesterases show activation followed by inhibition with substrate concentration. Biochem. J..

[57] Stojan J, Brochier L, Alies C, Colletier JP, Fournier D (2004). Inhibition of *Drosophila*
*melanogaster* acetylcholinesterase by high concentrations of substrate. Eur. J. Biochem..

[58] Szegletes T, Mallender WD, Thomas PJ, Rosenberry TL (1999). Substrate binding to the peripheral site of acetylcholinesterase initiates enzymatic catalysis. Substrate inhibition arises as a secondary effect. Biochemistry.

[59] Vandervalk LC, Nasr M, Dosdall LM (2014). New miticides for integrated pest management of *Varroa*
*destructor* (Acari: Varroidae) in honey bee colonies on the Canadian prairies. J. Econ. Entomol..

[60] RCore Team. *R:**A**Language and Environment for Statistical Computing*. (R Foundation for Statistical Computing, 2022).

[61] RStudio Team. *RStudio: Integrated Development Environment for R*. (RStudio, PBC, 2022).

