# Supplementary material for: Effects of dialkoxybenzenes against Varroa destructor and identification of 1-allyloxy-4-propoxybenzene as a promising acaricide candidate
Source: Sci Rep. 2023 Jul 11;13:11195. doi: 10.1038/s41598-023-38187-6 (PMC10336134; doi:10.1038/s41598-023-38187-6)
Supplement: Supplementary file 1 — Supplementary Information. [file 41598_2023_38187_MOESM1_ESM.pdf]

Supplemental Information

Effects of dialkoxybenzenes against *Varroa destructor* and identification of 1-allyloxy-4-propoxybenzene as a promising acaricide candidate

Soniya Dawdani [1†] Marissa O'Neill [1†], Carlos Castillo [2], Jorge E. Macias Sámano [1], Heather Higo [1], Abdullah Ibrahim [2], Stephen F. Pernal [2], and Erika Plettner [1*]

[1] Department of Chemistry, Simon Fraser University, 8888 Univ. Dr., Burnaby, B. C., V5A 1S6, Canada
[2] Agriculture and Agri-Food Canada, Beaverlodge Research Farm, P.O. Box 29, Beaverlodge, Alberta, T0H 0C0, Canada

* Author for correspondence:
Erika Plettner
Tel: 778-782-3586, E-mail: plettner@sfu.ca

† Equal contribution

1. Supplemental methods

1.1 Extraction of acetylcholinesterase (AChE) from invertebrates

Isolation of AChE from Apis mellifera

Several hundred honey bees were collected off combs of multiple hives from Honey Bee Zen apiary (New Westminster, BC, Canada). The bees were anesthetized with CO_2 before being frozen and stored at -80˚C. The heads were removed from 280 nurse bees, and their antennae discarded. Tissues and buffer solutions were kept on ice for as long as possible during the extraction. The phosphate buffer used contained 1% Triton-X-100 (v/v) to dissolve membrane-bound AChE, and filter-sterilized aprotinin (0.1µ g/mL) to protect isolated proteins from degradation. The bee heads were thoroughly homogenized in 20 mL of phosphate buffer. The homogenate was then centrifuged at 15,000 × g for 40 min, 4ºC. The supernatant was removed and centrifuged for an additional 20 min at 20,000 × g, 4ºC. The supernatant was removed and filter-sterilized (0.45 µm), and 10% sterile glycerol was added before the solution was aliquoted and flash frozen. The AChE isolate was then stored at -80˚C until it was used in assays.

The concentration of the isolated protein solution was determined using the Bradford Assay. Bovine serum albumin (BSA) was obtained from Sigma-Aldrich Canada Co. (Oakville, Ontario), and used as a standard. Eleven concentrations of BSA were prepared between 0.238 µg/mL and 14.29 µg/mL, and their absorbance was measured at 595 nm in the presence of Bradford Reagent. Absorbance over concentration was plotted, and the linear part of the curve ($R^2 > 0.99$) was determined. This linear portion was used to calculate the concentration of protein isolate based on its absorbance in the presence of Bradford Reagent. The concentration was calculated as 22.25 µg/mL; the value which was used to determine Specific Activity of Honey Bee AChE assays.

Isolation of AChE from Varroa mites

Adult female *Varroa* mites were collected from mite infested comb from Langley, BC, Canada. Mites were removed from emerging bees or from brood cells with a fine paint brush. All the mites were directly placed in Eppendorf tubes, flash frozen and stored at -80˚C until use. We used 380 mites in this assay.

Whole varroa mites were used for extracting *Varroa* AChE (*Vd*AChE) because of very small size of the mite. The mites and extracts were kept on ice. First, varroa were homogenized in a sterilized Eppendorf tube using a sealed glass pipette as a pestle. Phosphate buffer pH 7.6 in 1% Triton X-100 was used as a homogenizing solution (5 µl homogenizing solution per mite). The homogenate in the Eppendorf tube was sonicated (Branson Ultrasonic, fitted with a micro tip) and centrifuged at 15000 × g for 5 min at 4˚C for the pellets to settle down. The supernatant was transferred using micropipettes into sterile Eppendorf tubes. The first extraction was done with one third of the total homogenizing solution. The above procedure was repeated for the second extraction and the pellets were placed in the 3 mL glass homogenizer for the last extraction. All the supernatants were collected in one tube and centrifuged again at 15000 × g for 5 min at 4 ˚C. The brown extract solution was aliquoted (100 µL) in sterile Eppendorf tubes, by filtration

through a 0.45 µm filter. Aliquots were flash frozen in liquid nitrogen and stored at -40° C until further analyses.

Bradford assay: Ten concentrations (0 to 20 µg/ml) of BSA were prepared from 0.1 mg/ml of stock BSA to determine the unknown concentration of the varroa protein extract. Absorbance was measured at 595 nm. Varroa mite extract (10 µl) at dilutions (1:2, 1:4 and 1:10) were used to give absorbance within the BSA absorbance range. The average of protein concentration calculated from all three dilutions was 6.86 µg/mL. This value was used to calculate specific AChE activity of varroa extracts.

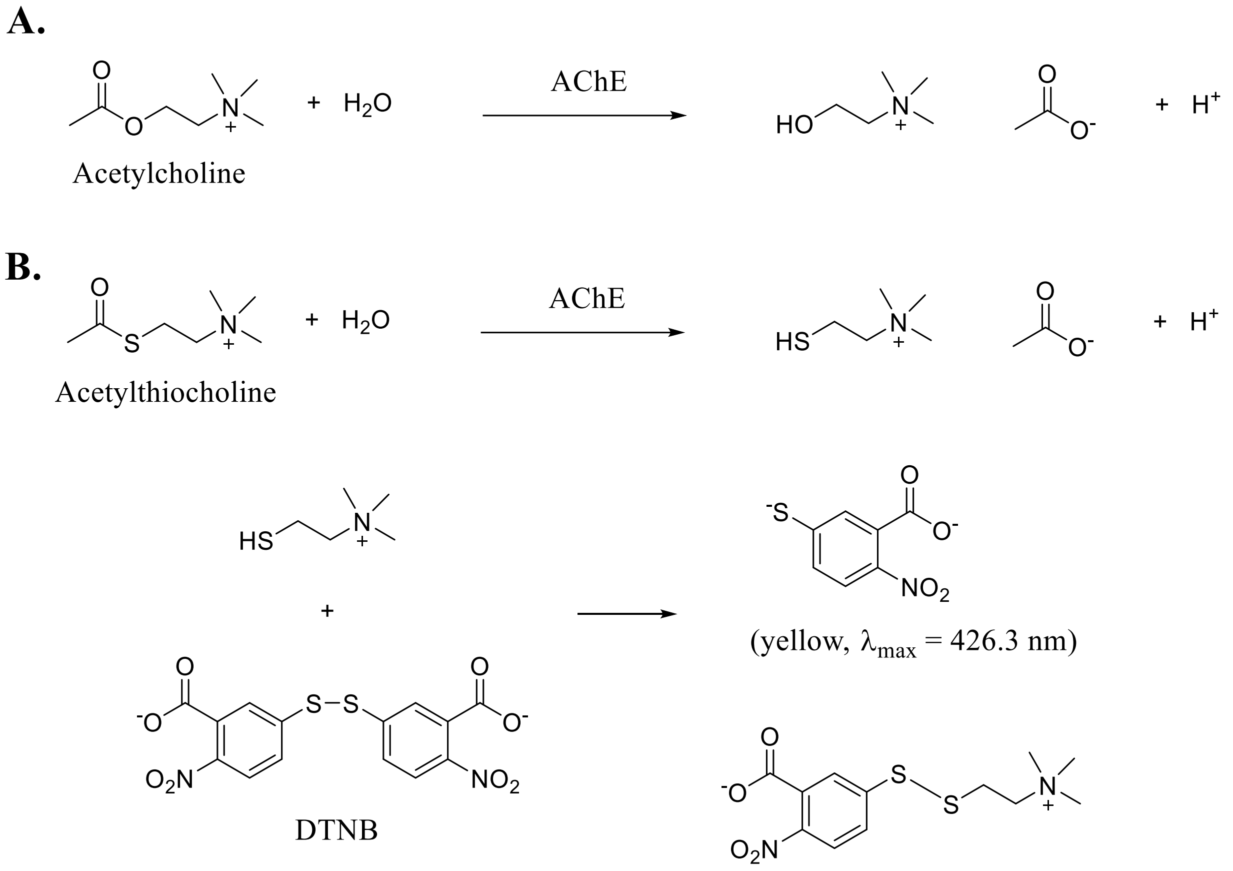

Figure S1. A. Reaction catalyzed by acetylcholinesterase (AChE). **B.** Coupled reactions used to detect AChE activity in the Ellman assay.

Determination of the Extinction Coefficient (ε)

We used 2-mercaptoethanol (βME) as a reducing agent for DTNB to determine ε. A 10 mM stock solution of βME in phosphate buffer was prepared. 892 µL of 100 mM Phosphate buffer was combined in a cuvette with 100 µL of 10 mM DTNB and 8 µL of 10 mM βME. The solution was inverted, and absorbance was measured from 300-500 nm. Three runs were performed, and

the average wavelength which gave the highest absorbance of TNB^{2-} was 426.3 nm. Consequently, a λ_{max} of 426 nm was used in all AChE assays. The absorbance was then measured at 426 nm for varying βME concentrations from 0.045 mM to 0.005 mM. Three replicate measures of each concentration were performed, and the absorbance was averaged. Absorbance was plotted against βME concentration. The linear portion ($R^2 > 0.99$) of the data points yielded an ε value of 11792 $cm^{-1}M^{-1}$. This extinction coefficient was therefore used to calculate the concentration of product formation for all AChE assays conducted.

Optimization of Tween 20 Concentration:

Optimization of Tween 20 concentration in our compound stocks was necessary to prevent the precipitation of the compounds during assay runs. Twelve Tween 20 concentrations between 0.02% -1.4% (v/v) were tested over the course of 5 minutes to ensure a lack of precipitation. For each Tween 20 concentration, 0.5 mM, 1 mM, and 3 mM of test compound were tested, and three replicates were performed. The optimal Tween 20 concentration was 1.1% in the assay mixture (stock inhibitor concentration of 55%). This concentration did not result in any precipitation over the course of 5 minutes for 0.5 mM, 1 mM, and 3 mM of any test compound.

1.2. Field trials

Placement of release devices

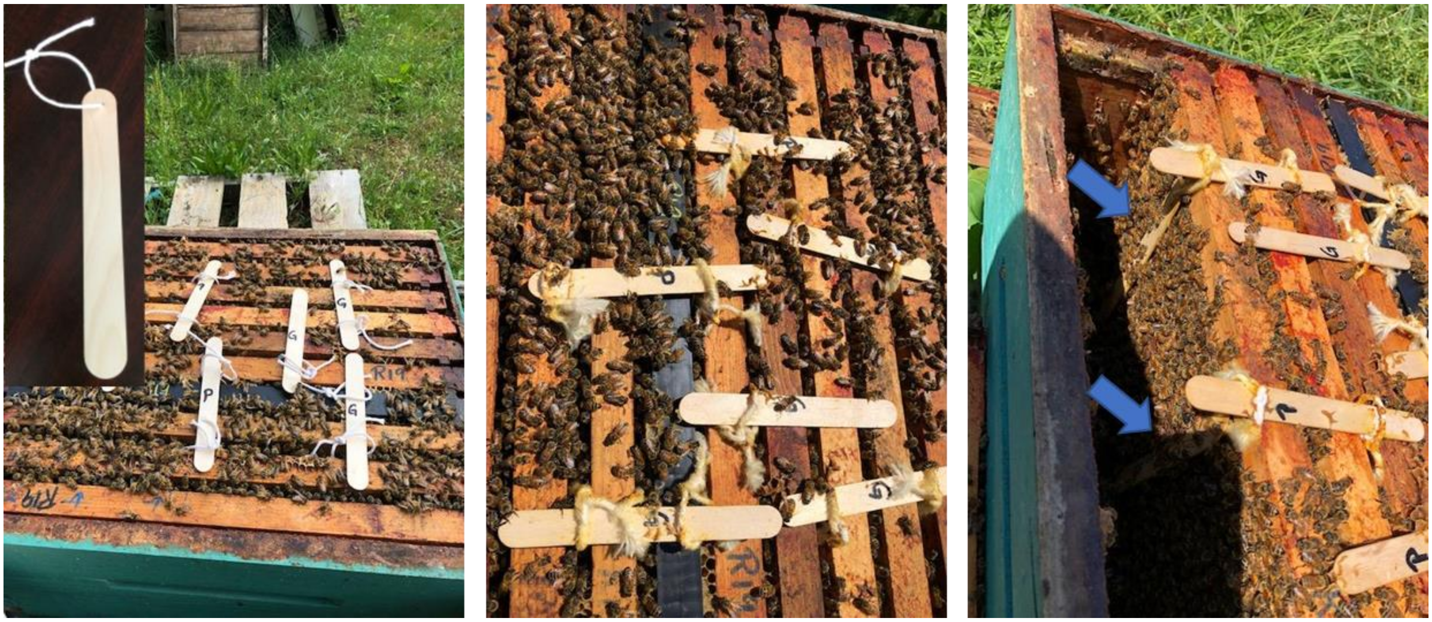

Figure S2. Placement of release devices with **3c**{*3,6*} (treatment) or no compound (control). The craft sticks placed horizontally across the top bars of the frames were used to suspend the treatment devices (insert on left) between the combs within the frame inter-spaces (see photo on right, blue arrows).

Analyses of Porapak devices. Porapak devices were retrieved from the colonies, wrapped individually with Al foil, placed in plastic Ziplock bags and taken to SFU Burnaby for analysis.

Devices were stored at -70°C until extraction. Devices were extracted with HPLC-grade hexane:EtOAc 4:1 with 1,4-dimethoxybenzene (Sigma), 20 ng/µL (= 2 mg/100 mL) as internal standard. Each Porapak-containing pipette was placed above a pipette fitted with a glass wool plug and loaded with silica gel (~ 2 cm high) and a layer of Na_2SO_4 (as drying agent). Solvent (5 mL) was added to the Porapak pipette and allowed to run through both pipettes prior to collection. The silica gel was used to remove any polar compounds deposited by the bees and the drying agent removed any moisture captured in the Porapak device. The volume of the solvent collected was determined.

Samples were run on a Perkin Elmer Clarus 690 GC interfaced with a Clarus SQ8T MS, equipped with a Velocity-5 30 m column (0.25 mm i. d., 0.2 µm film thickness). The GC was programmed 80°C (5 min), 10°/min to 250°C (10 min). Samples were injected using an automatic injector (1 µL), operated in splitless mode. Mass spectra were collected in EI+ mode from 50-350 amu, from 5 min to 25 min. The scan time was 0.35 s and the inter scan delay was 0.05 s.

Calibration was done with a pure standard of **3c**{*3,6*} in the extraction solvent with internal standard.

Analyses of alcohol from washes. Two mL of alcohol from the wash was taken and diluted with 2 mL of distilled water. The mixture was extracted with 2×2 mL of solvent (see above hex:EtoAc 4:1 with 20 ng/µL internal standard). Combined organic phases were dried over Na_2SO_4 and the volume of dry extract was determined. Samples were run by GC-MS as described above for the Porapak devices.

Analyses of wax samples. To the wax collected from the hives (~ 1 g) was added 2 mL of isopropanol. The samples were left to extract overnight at 4°C. Next, the isopropanol layer was collected, diluted with 2 mL of water and extracted as described above for the washes.

Analyses of honey samples. To the honey (with wax cells) collected from the hives was added 2 mL of isopropanol. Samples were left to extract overnight at 4°C. The remaining steps were as described above for the other samples.

Supplemental results

Structure-activity study (2018)

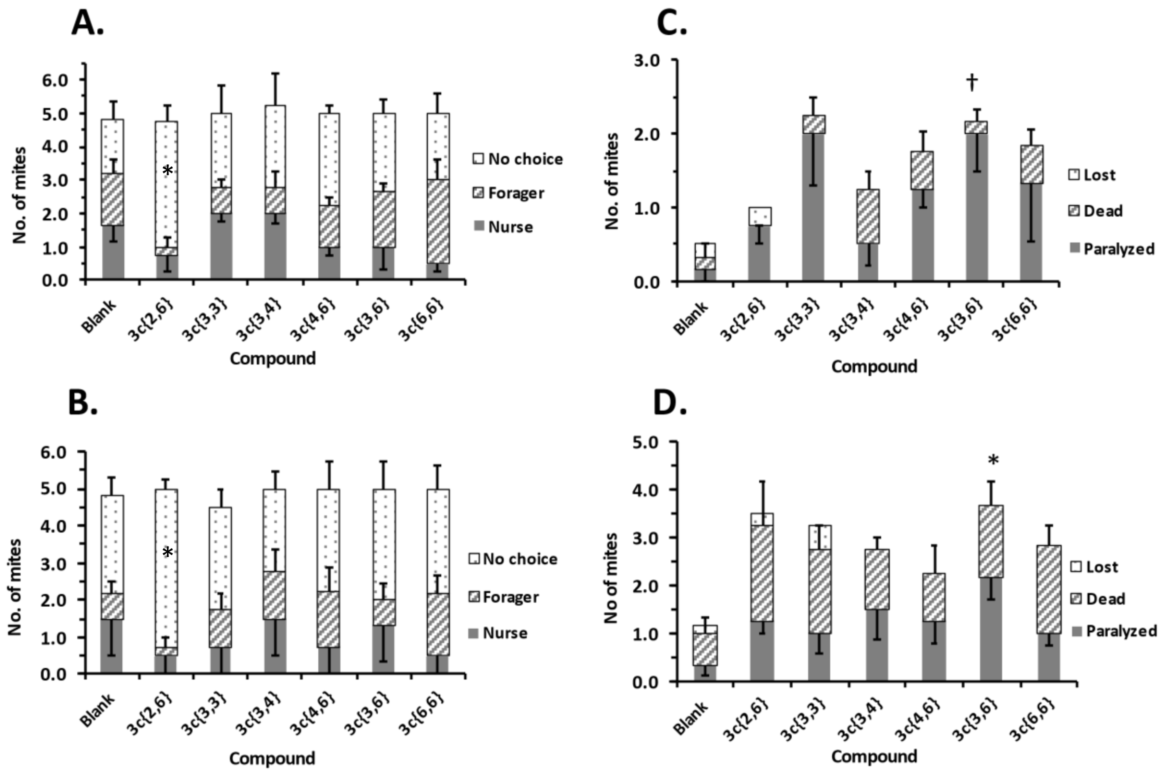

Figure S3. Results from a limited structure-activity survey of dialkoxybenzenes against varroa mites, done in 2018 (see text, under "Results Structure-activity relationship). **A.** Host choice after 3 hours of treatment: nurse (solid), forager (hatched), no choice (stippled). **B.** Host choice after 5 hours of treatment. There were no significant differences in host choice, except for **3c**{*2,6*} in which mites not having made a choice were significantly higher than in all others (Kruskal-Wallis, $p < 0.05$). **C.** Number of mites lost (stippled), dead (hatched) or paralyzed (solid gray) after 3 hours of treatment. † Compound **3c**{*3,6*} differed marginally from the blank with regard to paralysis and death+paralysis (Kruskal-Wallis, $p < 0.1$) **D.** Number of mites lost (stippled), dead (hatched) or paralyzed (solid gray) after 5 hours of treatment. * Compound **3c**{*3,6*} differed significantly from the blank with regard to paralysis and death+paralysis (Kruskal-Wallis, $p < 0.05$).

Structure-activity study 2020

Please see the main text for the experiments done. Acaricidal activity at 5 h was analyzed by quantitative structure-activity relationship (QSAR) in Molecular Operating Environment (Chemical Computing). The following descriptors were computed and validated: dipole moment, total energy, electrostatic energy, angular energy, non-bonding energy, van der Waals energy, highest occupied molecular orbital, lowest unoccupied molecular orbital, total accessible surface

area (ASA), positive ASA, negative ASA, hydrophobic ASA, polar ASA and van der Waals surface area.

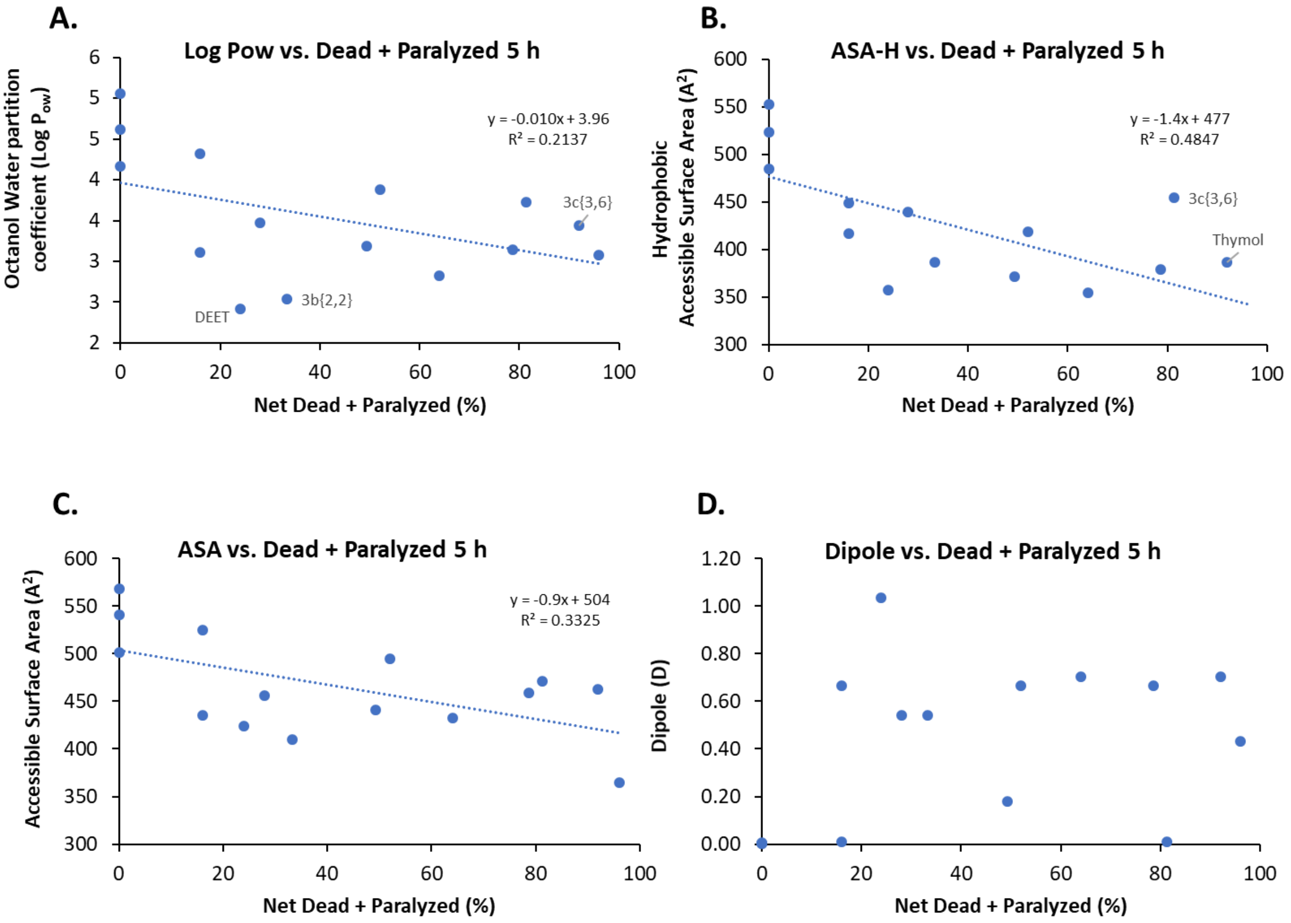

Figure S4. Results from quantitative structure-activity relationship (QSAR) analysis. **A.** Correlation of Log P_{ow} (octanol water partition coefficient) *vs*. activity (percentage of net dead + paralyzed mites at 5 h). **B.** Correlation of the hydrophobic accessible molecular surface area *vs*. activity. **C.** Correlation of the total accessible molecular surface area *vs*. activity. **D.** Plot of the dipole *vs*. activity (there was no correlation).

<u>Volatility assay</u>

For the evaporation experiment, 2.3 µL of the compound stock solution (0.5 µmol/µL) was dispensed onto the parafilm square (15 mm x 15 mm). The parafilm square was already placed at the bottom of the 4-dram vial before addition of compound solution. The vial was sealed with a plug-type rubber stopper. The vial setup was placed in the incubator (37 ^{o}C) for 3 hours. For the gas chromatography-mass spectrometry (GC-MS) run, 1 mL of the headspace was collected and injected. The GC-MS instrument was a Perkin Elmer Claurs 690 GC interfaced with a Perkin Elmer Clarus SQ8T MS, and equipped with a Velocity-5 30 m column (0.25 mm i. d., 0.2 µm

film thickness). Please see above for MS parameters and calibration. Samples were injected manually through a 1 mL gastight syringe.

A.

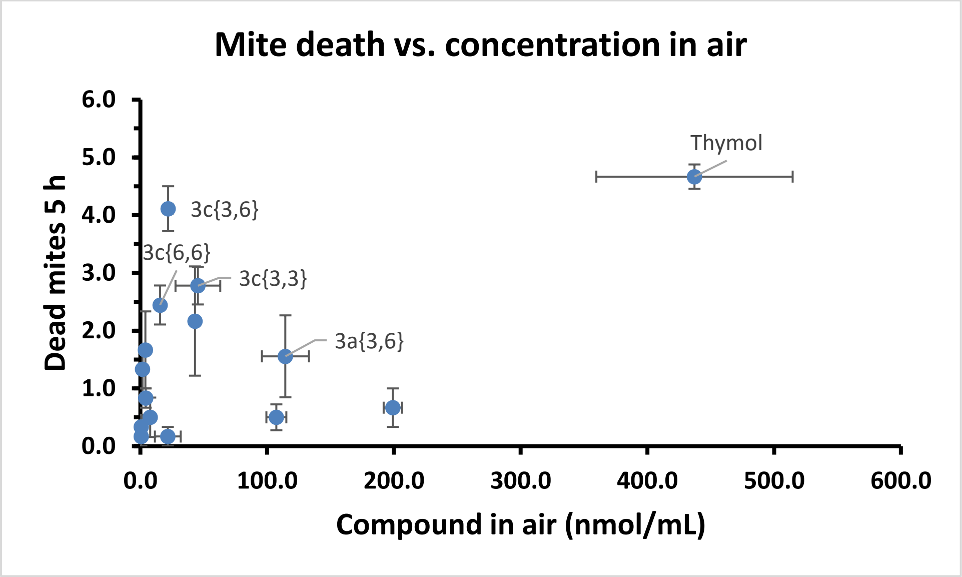

B.

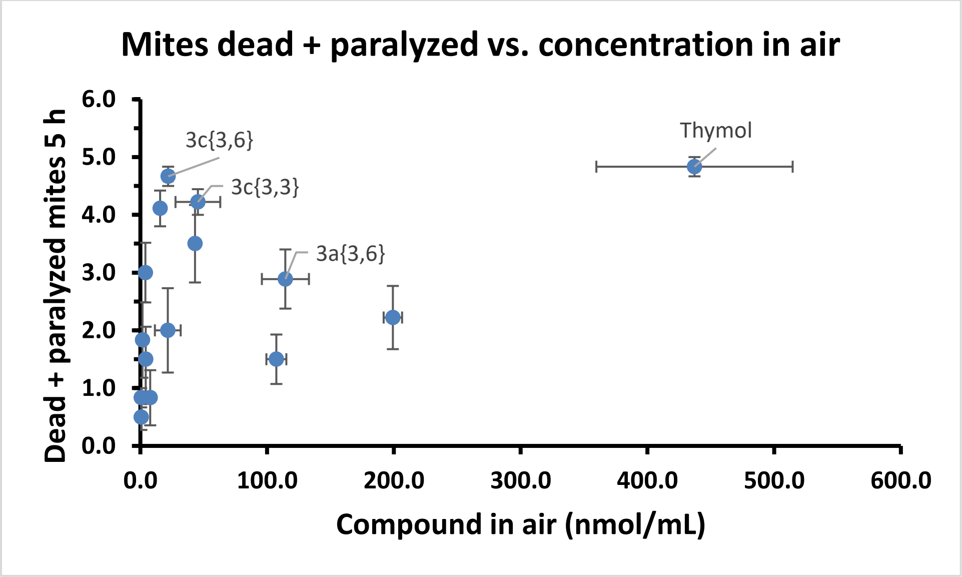

Figure S5. Plots of activity *vs*. the amount of compound in the air. **A.** Number of dead mites in the SAR assay after 5 h of treatment (see Fig. 2) *vs*. amount of compound in air in the volatility assay. **B.** Number of dead + paralyzed mites after 5 h of treatment *vs*. amount of compound in air in the volatility assay.

Table S1. Data table for Structure-Activity Relationship (SAR) 2020

Compound	N	3 Hours					5 Hours				
		Dead	Paralyzed	Glass	Abdomen	Not abdomen	Dead	Paralyzed	Glass	Abdomen	Not abdomen
Control	12	0.00 ± 0.00	0.33 ± 0.19	1.67 ± 0.40	2.67 ± 0.36	0.58 ± 0.15	0.25 ± 0.18	0.58 ± 0.15	2.00 ± 0.49	2.25 ± 0.48	0.58 ± 0.19
Thymol	6	2.50 ± 0.56	1.17 ± 0.31	4.67 ± 0.21	0.00 ± 0.00	0.33 ± 0.21	4.67 ± 0.21	0.17 ± 0.17	4.83 ± 0.17	0.00 ± 0.00	0.17 ± 0.17
DEET	6	0.67 ± 0.33	1.33 ± 0.33	2.00 ± 0.58	2.17 ± 0.40	0.67 ± 0.33	1.33 ± 0.42	0.50 ± 0.34	1.83 ± 0.70	2.00 ± 0.68	0.83 ± 0.31
**3c**{*2,6*}	6	0.17 ± 0.17	0.33 ± 0.21	2.50 ± 0.62	0.83 ± 0.65	1.17 ± 0.40	2.17 ± 0.95	1.33 ± 0.49	3.33 ± 0.67	0.83 ± 0.40	0.83 ± 0.31
**3c**{*3,6*}	9	3.11 ± 0.31	0.89 ± 0.31	3.33 ± 0.33	0.44 ± 0.18	0.89 ± 0.31	4.11 ± 0.39	0.56 ± 0.24	4.00 ± 0.37	0.22 ± 0.22	0.67 ± 0.24
**3c**{*4,6*}	6	0.33 ± 0.21	1.17 ± 0.17	2.50 ± 0.34	1.17 ± 0.17	1.17 ± 0.17	1.67 ± 0.67	1.33 ± 0.33	3.33 ± 0.21	0.67 ± 0.33	0.83 ± 0.31
**3c**{*n5,6*}	6	0.83 ± 0.40	0.67 ± 0.21	1.67 ± 0.67	1.83 ± 0.65	1.33 ± 0.21	0.83 ± 0.17	0.67 ± 0.33	1.67 ± 0.61	1.67 ± 0.42	1.67 ± 0.42
**3c**{*6,6*}	9	0.78 ± 0.28	1.56 ± 0.47	3.67 ± 0.37	0.56 ± 0.29	0.56 ± 0.24	2.44 ± 0.34	1.67 ± 0.50	4.44 ± 0.29	0.11 ± 0.11	0.44 ± 0.24
**3c**{*3,3*}	9	0.89 ± 0.26	1.78 ± 0.49	3.33 ± 0.44	1.00 ± 0.33	0.56 ± 0.24	2.78 ± 0.32	1.44 ± 0.29	4.22 ± 0.28	0.22 ± 0.15	0.44 ± 0.24
**3c**{*3,4*}	6	0.00 ± 0.00	0.33 ± 0.21	1.67 ± 0.49	1.67 ± 0.42	1.67 ± 0.33	0.50 ± 0.34	0.33 ± 0.21	1.67 ± 0.49	1.83 ± 0.54	1.33 ± 0.76
**3c**{*3,n5*}	6	0.00 ± 0.00	0.33 ± 0.21	0.67 ± 0.21	2.00 ± 0.26	2.33 ± 0.21	0.33 ± 0.21	0.50 ± 0.22	1.00 ± 0.26	2.00 ± 0.52	2.00 ± 0.45
**3c**{*4,n5*}	6	0.00 ± 0.00	0.17 ± 0.17	1.00 ± 0.37	3.17 ± 0.31	0.83 ± 0.31	0.17 ± 0.17	0.33 ± 0.21	1.33 ± 0.49	2.50 ± 0.34	1.00 ± 0.45
**3a**{*3,6*}	9	0.00 ± 0.00	1.33 ± 0.33	4.22 ± 0.28	0.44 ± 0.29	0.33 ± 0.17	1.56 ± 0.71	1.33 ± 0.47	4.11 ± 0.26	0.33 ± 0.17	0.56 ± 0.18
**3b**{*3,6*}	6	0.17 ± 0.17	0.50 ± 0.22	2.33 ± 0.80	1.50 ± 0.43	1.33 ± 0.42	0.17 ± 0.17	1.50 ± 0.72	2.67 ± 0.67	1.33 ± 0.42	1.17 ± 0.48
**3b**{*2,2*}	9	0.11 ± 0.11	0.78 ± 0.32	2.89 ± 0.63	1.00 ± 0.44	1.11 ± 0.39	0.67 ± 0.33	1.56 ± 0.34	2.56 ± 0.63	1.22 ± 0.43	1.11 ± 0.31
**3c**{*2,3*}	6	0.17 ± 0.17	0.67 ± 0.33	1.33 ± 0.21	1.50 ± 0.50	1.83 ± 0.40	0.50 ± 0.22	1.00 ± 0.26	2.33 ± 0.56	1.67 ± 0.21	0.83 ± 0.48

Significantly larger than the control ($p < 0.05$)

Significantly lower than the control ($p < 0.05$)

Time	Compound	Control	**3c**{*3,6*}					
		0 ng	0.01 ng	0.1 ng	1 ng	10 ng	100 ng	1000 ng
2 Hours	Dead	0.00 ± 0.00	0.00 ± 0.00	0.00 ± 0.00	0.00 ± 0.00	0.00 ± 0.00	0.00 ± 0.00	0.40 ± 0.24
	Paralyzed	0.00 ± 0.00	0.00 ± 0.00	0.80 ± 0.37	0.80 ± 0.37	0.00 ± 0.00	0.60 ± 0.24	1.00 ± 0.00
	Glass	0.39 ± 0.10	0.60 ± 0.24	1.20 ± 0.37	1.60 ± 0.40	0.40 ± 0.24	1.20 ± 0.37	1.40 ± 0.24
	Abdomen	3.74 ± 0.21	3.00 ± 0.55	1.80 ± 0.49	1.80 ± 0.49	3.20 ± 0.49	2.40 ± 0.51	2.00 ± 0.32
	Not abdomen	0.83 ± 0.21	1.20 ± 0.49	1.80 ± 0.37	1.60 ± 0.40	1.40 ± 0.40	1.20 ± 0.37	0.80 ± 0.37
4 Hours	Dead	0.00 ± 0.00	0.40 ± 0.24	1.00 ± 0.45	2.20 ± 0.37	1.00 ± 0.32	2.60 ± 0.24	3.40 ± 0.24
	Paralyzed	0.13 ± 0.07	0.40 ± 0.24	0.80 ± 0.20	0.40 ± 0.24	0.80 ± 0.37	1.20 ± 0.37	0.60 ± 0.24
	Glass	0.57 ± 0.15	1.20 ± 0.58	1.20 ± 0.58	2.60 ± 0.24	1.80 ± 0.37	3.40 ± 0.51	3.00 ± 0.32
	Abdomen	3.43 ± 0.23	3.00 ± 0.55	2.60 ± 0.68	1.40 ± 0.40	2.20 ± 0.66	0.80 ± 0.37	0.60 ± 0.24
	Not abdomen	0.87 ± 0.20	0.60 ± 0.24	0.80 ± 0.20	0.80 ± 0.37	1.00 ± 0.45	0.80 ± 0.37	1.20 ± 0.20
6 Hours	Dead	0.09 ± 0.06	1.80 ± 0.49	2.60 ± 0.60	3.60 ± 0.24	3.80 ± 0.37	4.20 ± 0.37	4.60 ± 0.24
	Paralyzed	0.30 ± 0.10	0.60 ± 0.24	0.60 ± 0.40	0.40 ± 0.24	0.60 ± 0.24	0.40 ± 0.24	0.00 ± 0.00
	Glass	1.30 ± 0.19	3.20 ± 0.58	3.00 ± 0.45	4.40 ± 0.24	3.80 ± 0.20	4.00 ± 0.45	4.40 ± 0.24
	Abdomen	2.65 ± 0.13	1.00 ± 0.32	1.80 ± 0.37	0.40 ± 0.24	0.40 ± 0.24	0.20 ± 0.20	0.40 ± 0.24
	Not abdomen	0.91 ± 0.21	0.60 ± 0.40	0.20 ± 0.20	0.20 ± 0.20	0.60 ± 0.24	0.60 ± 0.40	0.00 ± 0.00

Table S2. Data table for Direct Contact Assay with **3c**{*3,6*}. Data points are average +/- S. E. (5 replicates).

Time	Compound	Control	3c{6,6}					
		0 ng	0.01 ng	0.1 ng	1 ng	10 ng	100 ng	1000 ng
2 Hours	Dead	0.00 ± 0.00	0.00 ± 0.00	0.60 ± 0.40	0.00 ± 0.00	0.00 ± 0.00	0.00 ± 0.00	0.00 ± 0.00
	Paralyzed	0.00 ± 0.00	0.00 ± 0.00	0.20 ± 0.20	0.00 ± 0.00	0.00 ± 0.00	0.00 ± 0.00	0.00 ± 0.00
	Glass	0.39 ± 0.10	0.60 ± 0.40	0.00 ± 0.00	0.80 ± 0.20	1.20 ± 0.37	0.40 ± 0.24	1.00 ± 0.63
	Abdomen	3.74 ± 0.21	3.60 ± 0.68	2.80 ± 0.80	2.60 ± 0.24	2.00 ± 0.55	2.00 ± 0.55	1.60 ± 0.40
	Not abdomen	0.83 ± 0.21	0.80 ± 0.58	1.60 ± 0.51	1.60 ± 0.24	1.60 ± 0.24	2.60 ± 0.40	2.20 ± 0.37
4 Hours	Dead	0.00 ± 0.00	0.00 ± 0.00	0.00 ± 0.00	0.40 ± 0.24	0.20 ± 0.20	0.80 ± 0.20	1.20 ± 0.20
	Paralyzed	0.13 ± 0.07	0.20 ± 0.20	0.80 ± 0.20	0.60 ± 0.24	0.80 ± 0.20	0.20 ± 0.20	0.40 ± 0.24
	Glass	0.57 ± 0.15	0.60 ± 0.60	2.40 ± 0.24	1.60 ± 0.51	1.60 ± 0.40	2.20 ± 0.20	2.80 ± 0.37
	Abdomen	3.43 ± 0.23	1.80 ± 0.37	0.80 ± 0.20	1.40 ± 0.51	1.00 ± 0.32	1.40 ± 0.24	1.40 ± 0.24
	Not abdomen	0.87 ± 0.20	2.60 ± 0.51	1.80 ± 0.20	1.80 ± 0.66	2.40 ± 0.24	1.20 ± 0.37	0.80 ± 0.24
6 Hours	Dead	0.09 ± 0.06	0.20 ± 0.20	1.00 ± 0.55	1.60 ± 0.40	1.00 ± 0.34	1.20 ± 0.20	2.40 ± 0.24
	Paralyzed	0.30 ± 0.10	0.40 ± 0.24	0.60 ± 0.24	0.40 ± 0.24	0.60 ± 0.24	0.60 ± 0.24	0.80 ± 0.20
	Glass	1.30 ± 0.19	1.60 ± 0.51	2.60 ± 0.51	3.00 ± 0.32	2.00 ± 0.55	2.40 ± 0.40	2.80 ± 0.49
	Abdomen	2.65 ± 0.13	1.80 ± 0.20	1.60 ± 0.24	0.60 ± 0.24	1.80 ± 0.37	1.20 ± 0.20	0.80 ± 0.20
	Not abdomen	0.91 ± 0.21	1.60 ± 0.40	0.60 ± 0.40	1.20 ± 0.20	1.00 ± 0.32	1.40 ± 0.24	0.80 ± 0.20

Table S3. Data table for Direct Contact Assay with **3c**{*6*,*6*}. Data points are average +/- S. E. (5 replicates).

2. Testing of **3c**{*3,6*}, **3c**{*6,6*} and the 1:1 blend on Varroa mites by themselves

The assay was set up as described in the methods, in glass dishes with a 2×2 cm^2 piece of Parafilm in the lid. The Parafilm received the treatment in hexane (10 μL). Blanks received only hexane (10 μL). One mite was placed in the center of the dish, and the setup was incubated at 32°C, as described in the main article. Four doses were tested: 200 μg, 500 μg, 1.0 mg and 10 mg. Five mites were placed in each dish. Compounds **3c**{*3,6*} and **3c**{*6,6*} were tested by themselves and in a 1:1 blend. The results (Fig. S6) show that compound **3c**{*3,6*} is more active than **3c**{*6,6*} and that blending results in dilution of the more active compound.

4. Additional results

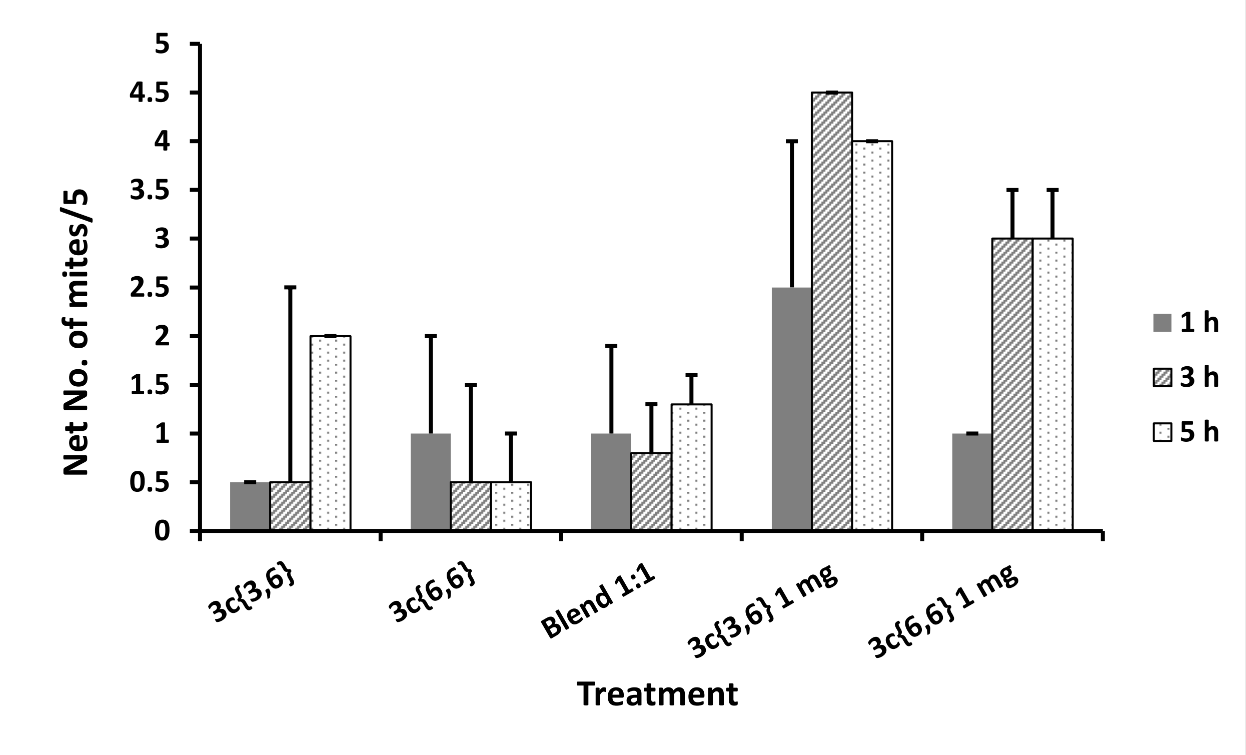

Figure S6. Effect of blending compounds **3c**{*3,6*} and **3c**{*6,6*} on the net number of mites paralyzed or dead. **A.** Experiment performed in 2018, with 500 μg of **3c**{*3,6*}, **3c**{*6,6*} and the 1:1 blend (500 μg each compound), as well as 1 mg of each pure compound. Bars represent the total of paralyzed + dead mites, after subtraction of the number obtained in the paired blank, averages ± S. E. of 4 replicates.

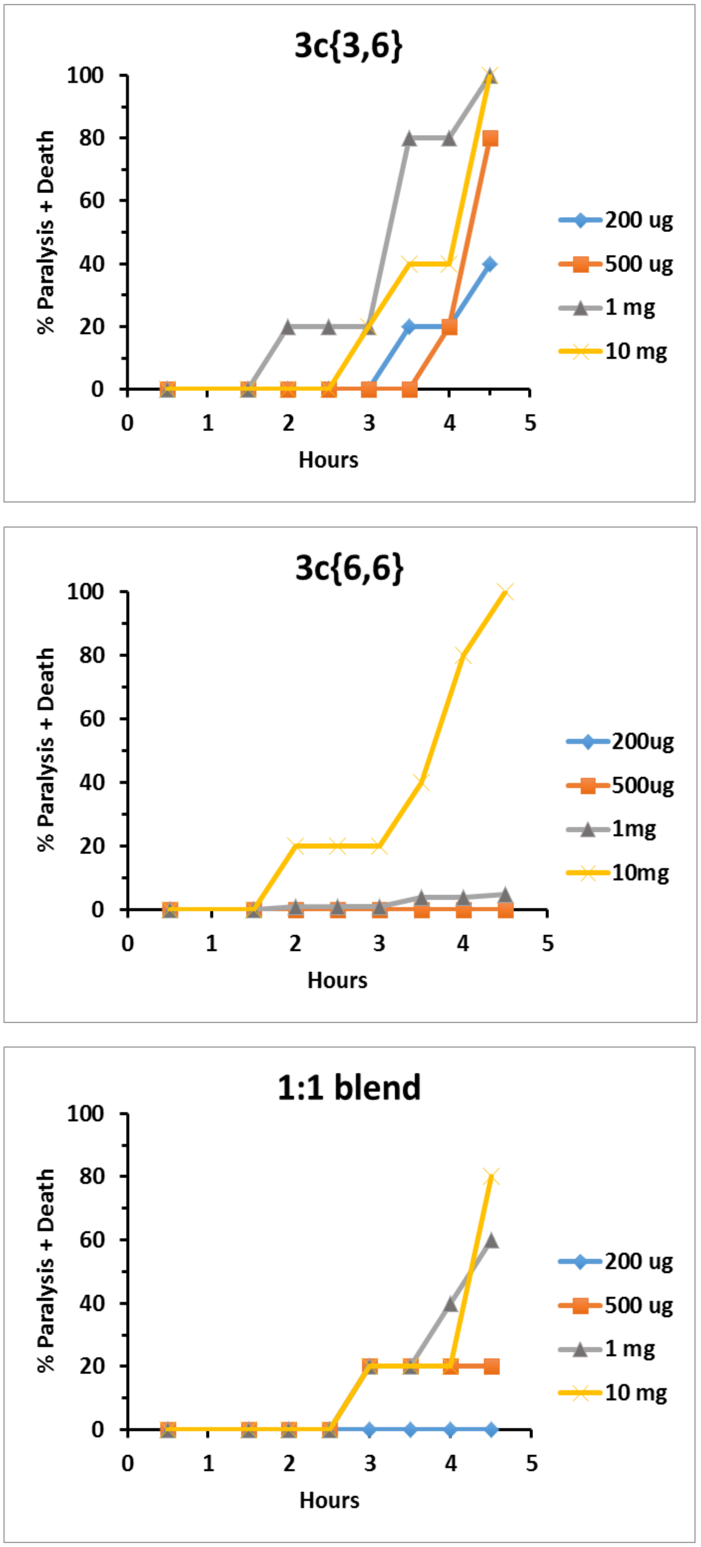

Figure S7. Test of mite paralysis and death for compounds **3c**{*3,6*} and **3c**{*6,6*} by themselves and in a 1:1 blend. Mites were in the test dishes by themselves (without bees).

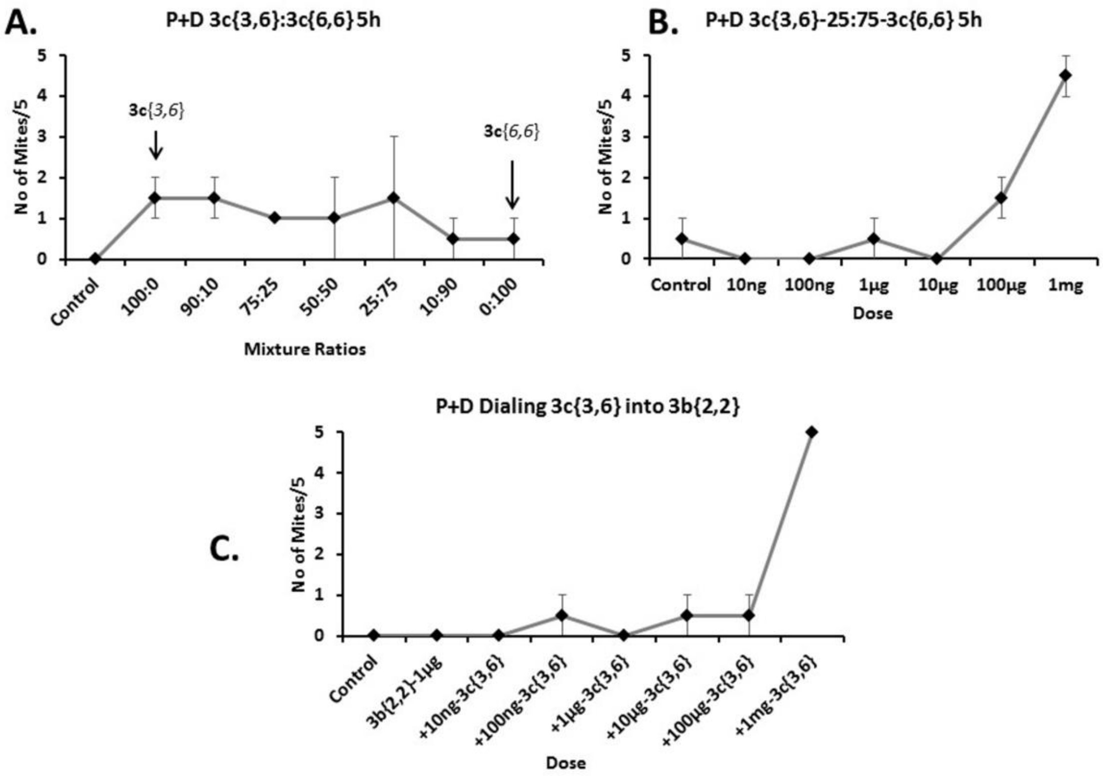

Figure S8. Activity of combinations of **3c**{*3,6*}, **3c**{*6,6*} and **3b**{*2,2*}. **A.** Dead + paralyzed mites for different combinations of **3c**{*3,6*} and **3c**{*6,6*}. **B.** Dose response for 1:3 mixture (by weight) of **3c**{*3,6*} and **3c**{*6,6*}. **C.** Combinations of **3b**{*2,2*} and **3c**{*3,6*}.

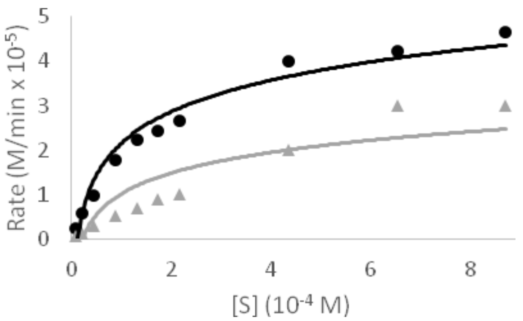
Rate (M/min x 10^{-5})
[S] (10^{-4} M)

a)

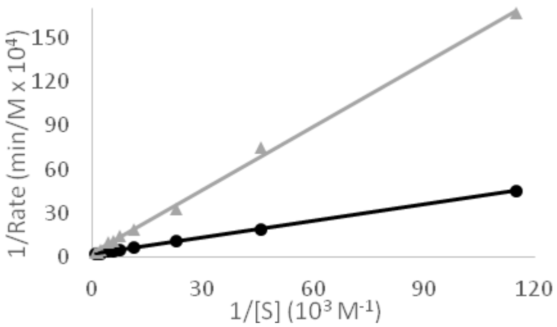
1/Rate (min/M x 10^{4})
1/[S] (10^{3} M^{-1})

b)

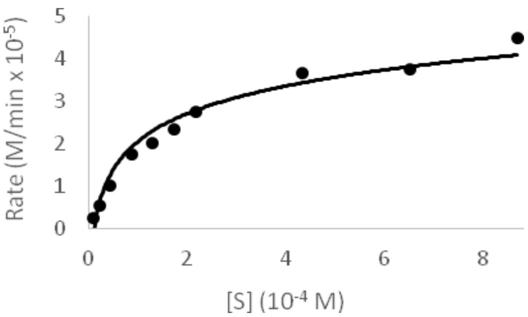
Rate (M/min x 10^{-5})
[S] (10^{-4} M)

c)

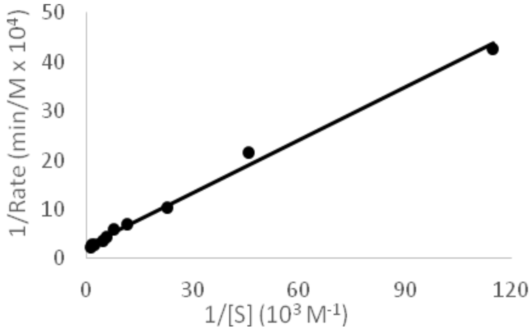
1/Rate (min/M x 10^{4})
1/[S] (10^{3} M^{-1})

d)

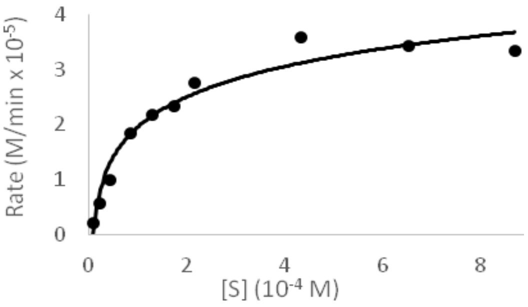
Rate (M/min x 10^{-5})
[S] (10^{-4} M)

e)

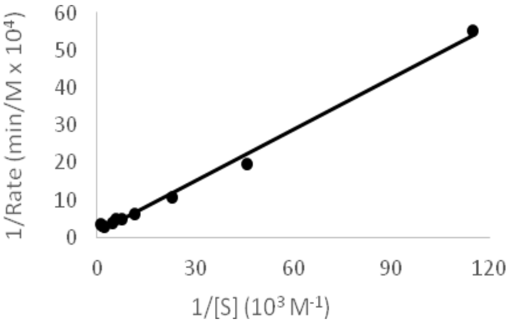
1/Rate (min/M x 10^{4})
1/[S] (10^{3} M^{-1})

f)

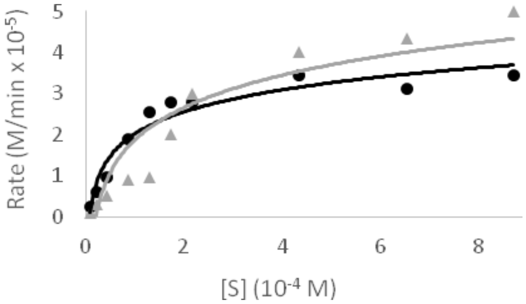
Rate (M/min x 10^{-5})
[S] (10^{-4} M)

g)

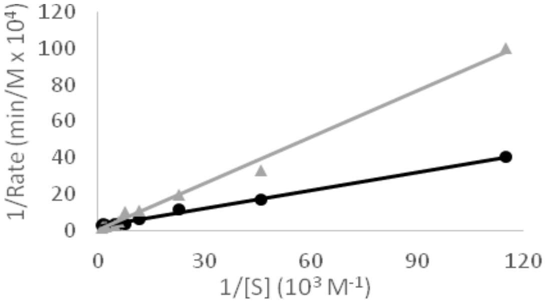
1/Rate (min/M x 10^{4})
1/[S] (10^{3} M^{-1})

h)

Figure S9. Kinetics of human AChE, *h*AChE. The set of graphs on the left, a, c, e, g show the Michaelis-Menten plots (rate *vs*. substrate concentration [S]); the set of graphs on the right, b, d, f, h show the corresponding Lineweaver-Burk plots (1/rate *vs*. 1/[S]). **a**) and **b)** *h*AChE in the presence of DEET. The black line shows the average rate *vs*. substrate concentration [S] (a) or the corresponding L-B plot for kinetics in the presence of 0 mM 0.5 mM and 1 mM DEET. The grey line shows the kinetics with 3 mM DEET which had a significant increase in K_m. **c)** and **d)** The average of *h*AChE in the presence of **3c**{*3,6*} with no significant differences between runs in the presence or absence of the compound. **e)** and **f)** The average of *h*AChE activity in the presence of **3c**{*6,6*} with no significant differences. **g)** and **h)** *h*AChE in the presence of **3b**{*2,2*}. The black line shows the average rate *vs*. substrate concentration for *h*AChE in the presence of 0 mM, 0.5 mM and 1 mM **3b**{*2,2*}. The grey line shows 3 mM **3b**{*2,2*} which had a significant increase in V_{max}.

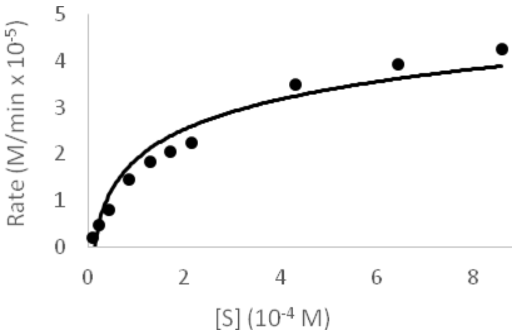
Rate (M/min x 10-5)
[S] (10-4 M)

a)

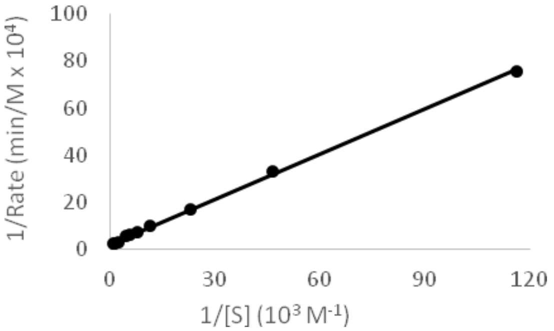
1/Rate (min/M x 104)
1/[S] (103 M-1)

b)

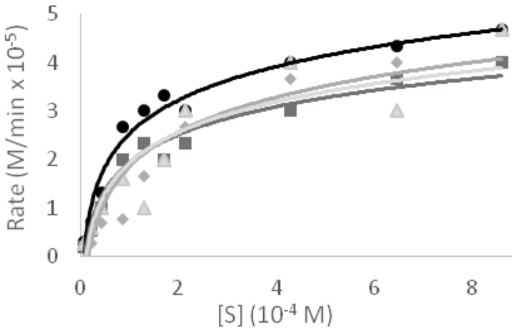
Rate (M/min x 10-5)
[S] (10-4 M)

c)

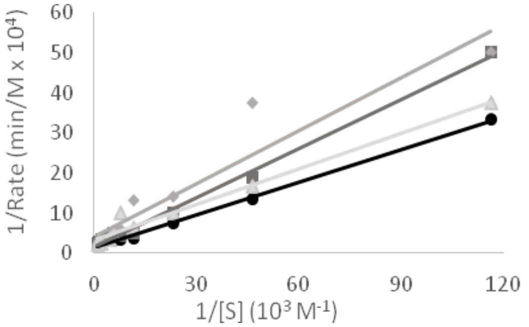
1/Rate (min/M x 104)
1/[S] (103 M-1)

d)

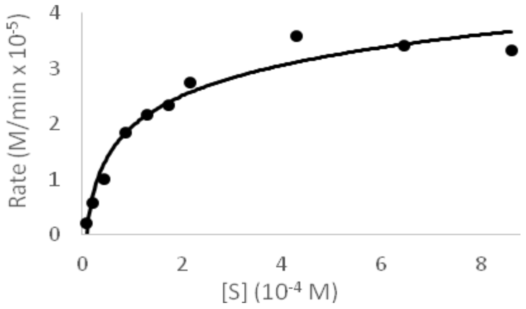
Rate (M/min x 10-5)
[S] (10-4 M)

e)

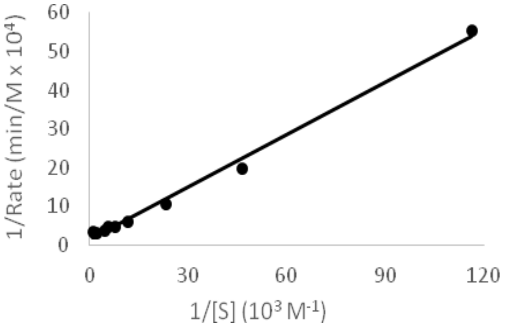
1/Rate (min/M x 104)
1/[S] (103 M-1)

f)

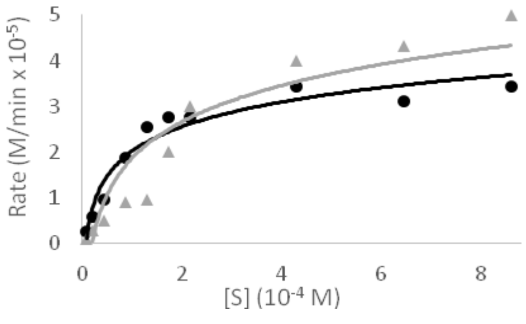
Rate (M/min x 10-5)
[S] (10-4 M)

g)

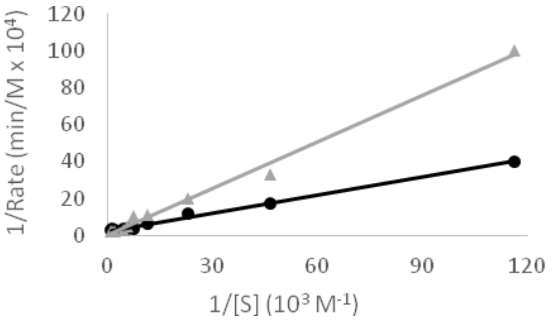
1/Rate (min/M x 104)
1/[S] (103 M-1)

h)

Figure S10. Kinetics of honey bee AChE, *Am*AChE. Left set of graphs, a, c, e, g show the Michaelis-Menten plots (rate *vs*. substrate concentration [S]); the right set of graphs, b, d, f, h show the Lineweaver-Burk plots (1/rate *vs*. 1/[S]). **a)** and **b)** *Am*AChE in the presence of DEET. There is no significant difference in kinetics of *Am*AChE at any concentration or DEET. **c)** and **d)** Kinetics of *Am*AChE in the presence of **3c**{*3,6*}; the black line represents 0 mM, darkest grey (square points) 0.5 mM, next darkest grey (diamond points) 1 mM, and lightest grey (triangle points) 3 mM of **3c**{*3,6*}. **e)** and **f)** *Am*AChE in the presence of **3c**{*6,6*}; no significant difference between treatments. **g)** and **h)** *Am*AChE in the presence of **3b**{*2,2*}. The black line represents the average of 0 mM, 0.5 mM, and 1 mM. The grey represents 3 mM which had a significant increase in V_{max}.

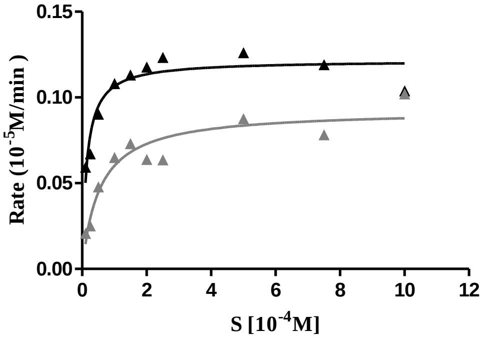

a)

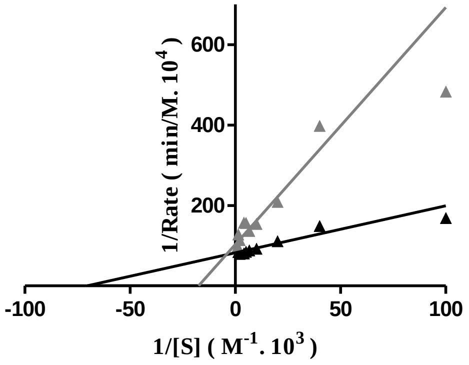

b)

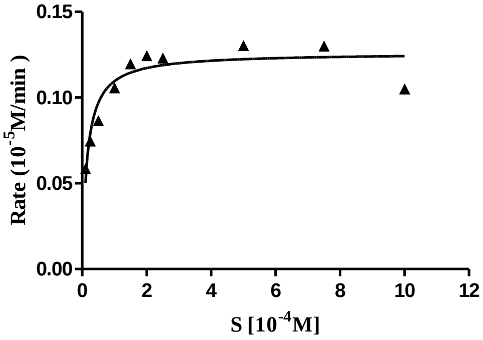

c)

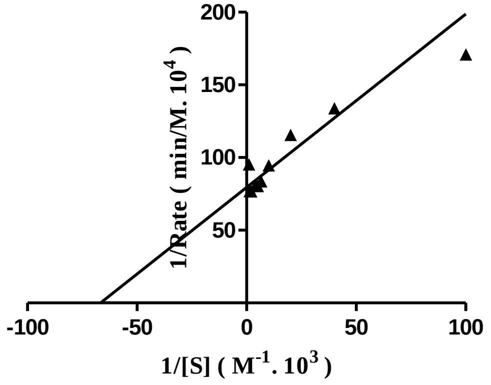

d)

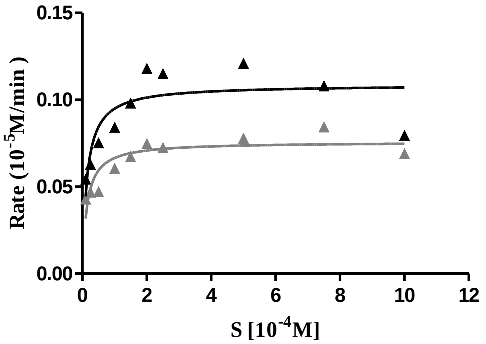

e)

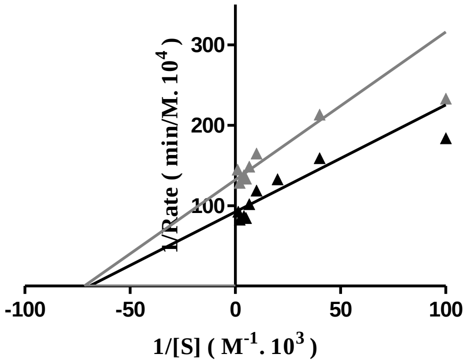

f)

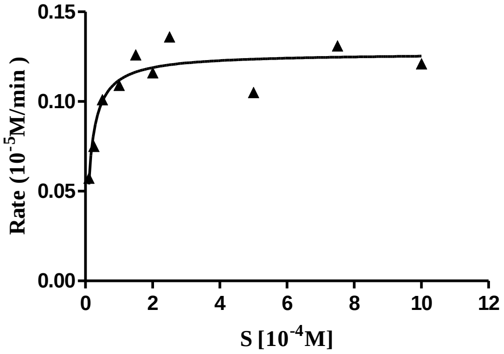

g)

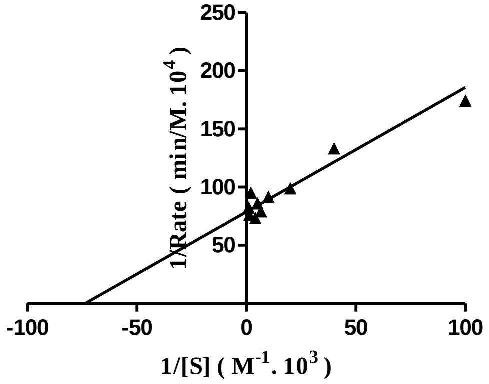

h)

Figure S11. Kinetics of AChE from varroa mites, *Vd*AChE. Left set of graphs, a, c, e, g show the Michaelis-Menten plots (rate *vs*. substrate concentration [S]); the right set of graphs, b, d, f, h show the Lineweaver-Burk plots (1/rate *vs*. 1/[S]). **a)** and **b)** The black lines show the average rate of *Vd*AChE *vs*. substrate concentration with 0 mM,0.5 mM and 1 mM of DEET (**a**) or the corresponding L-B plot (**b**). The grey lines show the data in the presence of 3 mM DEET, which gave a significant increase in K_M. **c)** and **d)** Average rate data of *Vd*AChE in the presence of 0.5 mM, 1 mM and 3mM **3c**{*3*,*6*}, between which there were no significant differences. **e)** and **f)** The black lines show kinetic data for *Vd*AChE in the absence of **3c**{*6*,*6*}. The grey lines show kinetic behavior for *Vd*AChE in the presence of 3 mM **3c**{*6*,*6*} which gave a significant decrease in V_{max} but no change in K_M. **g)** and **h)** Kinetics of *Vd*AChE in the presence of 3 mM **3b**{*2*,*2*} which did not have an effect.
